# A New High-Throughput Approach to Genotype Ancient Human Gastrointestinal Parasites

**DOI:** 10.1371/journal.pone.0146230

**Published:** 2016-01-11

**Authors:** Nathalie M. L. Côté, Julien Daligault, Mélanie Pruvost, E. Andrew Bennett, Olivier Gorgé, Silvia Guimaraes, Nicolas Capelli, Matthieu Le Bailly, Eva-Maria Geigl, Thierry Grange

**Affiliations:** 1 Institut Jacques Monod, CNRS, University Paris Diderot, UMR 7592, Epigenome and Paleogenome group, 15 rue Hélène Brion, 75205, Paris, Cedex 13, France; 2 University of Bourgogne Franche-Comte, CNRS UMR 6249 Chrono-environment, 16 route de Gray, 25030, Besançon cedex, France; University of Otago, NEW ZEALAND

## Abstract

Human gastrointestinal parasites are good indicators for hygienic conditions and health status of past and present individuals and communities. While microscopic analysis of eggs in sediments of archeological sites often allows their taxonomic identification, this method is rarely effective at the species level, and requires both the survival of intact eggs and their proper identification. Genotyping via PCR-based approaches has the potential to achieve a precise species-level taxonomic determination. However, so far it has mostly been applied to individual eggs isolated from archeological samples. To increase the throughput and taxonomic accuracy, as well as reduce costs of genotyping methods, we adapted a PCR-based approach coupled with next-generation sequencing to perform precise taxonomic identification of parasitic helminths directly from archeological sediments. Our study of twenty-five 100 to 7,200 year-old archeological samples proved this to be a powerful, reliable and efficient approach for species determination even in the absence of preserved eggs, either as a stand-alone method or as a complement to microscopic studies.

## Introduction

Infections with gastrointestinal helminths are a major public health concern. Soil-transmitted helminthiases affect about 2 billion people worldwide [1,2]. Estimates suggest that the nematode *Ascaris lumbricoides* infects nearly 1.2 billion people and *Trichuris trichiura* 800 million. *A*. *lumbricoides* infections, caused by ingesting food or soil contaminated with their eggs, result in roughly 60,000 deaths per year [1,2]. The eggs of the tapeworms (Cestoda) *Taenia sp*. and *Diphyllobothrium sp*., found in undercooked meat and fish respectively, develop larval stages that infect the muscles of the intermediate host [3]. *Echinococcus* infection can cause life-threatening cysticercoses. The flukes *Fasciola hepatica* and *Dicrocoelium sp*., helminths of the class of Trematoda, infect the liver of various mammals, including humans [4]. Liver infection by flukes in cattle and sheep causes severe economic losses [5]. In general, food- and soil-transmitted helminths produce a wide range of symptoms that include intestinal manifestations (diarrhea, abdominal pain), impaired physical growth, and general malaise and weakness that may affect working and learning capacities.

As high as these numbers are, the percentage of the human population infected with gastrointestinal helminths in the past was likely to have been even higher due to poor hygiene and insufficient medical treatment, but little is known of the past infection rates of various parasites. Owing to the impact of parasites on public health described above, there is a genuine interest of archaeologists and historians to evaluate the health status of ancient societies and populations through the analysis of the prevalence of parasites in archaeological sites. Indeed, gastrointestinal helminths release their eggs into the intestine, which then pass into the environment to resume their life cycle. These eggs are sometimes preserved over time in the sediments of archaeological sites and can be found in mummified tissues, in and under human and animal skeletons in burials, but also in latrines, cesspits and coprolites [6].

Taxonomic assignment of eggs is achieved through microscopic analysis using visually detectable differences in their size, shape, color, and opercula (for review, see [7]). This morphological approach has revealed interesting insights into the health status of past populations and individuals, their diet, and their contact with wild and domestic animals (e.g., [6,8,9]). Nevertheless, taphonomic processes, i.e., the processes by which biological material decays, can lead to the total degradation of eggs, giving rise to false-negative results [10]. Its outreach is also limited since morphological criteria often do not allow the discrimination between species or even genera (e.g., *Taenia sp*. and *Echinococcus sp*.). Assignment of archeological material to the species level, however, will permit a new level of anthropological and biological investigations.

The genetic analysis of DNA from these and other parasites preserved in eggs or sediments can overcome some of these limitations and yield results at a higher level of resolution. A paleogenetic approach would be restricted only by poor DNA preservation, which must be taken into account in the experimental design. Several studies have analyzed archeological remains using a polymerase chain reaction (PCR) approach targeting DNA fragments from 150 bp to 600 bp long (e.g., [11]). The prerequisite of such studies is that the present-day genetic diversity of the parasites is known and similar to that of the past. Moreover, these studies limit their PCR primer design to the parasites previously identified in the sample and still required microscopic identification of parasite eggs, thus excluding the identification of parasites that had escaped visual detection. Metagenomic studies would allow the identification of highly divergent or even unknown parasites [12], but the costs of such studies are often too high to be routinely used to answer archeological questions.

In order to improve the utility of PCR-based parasite identification studies, we have developed a cost-effective high-throughput approach tailored to the needs of archeologists, i.e., the analysis of relatively large numbers of archaeological remains without the need of prior microscopic analysis. This method uses optimized DNA extraction techniques from sediments and adapts the “*aMPlex Torrent”* approach to the analysis of ancient parasite DNA preserved in archaeological sediments of various origins (such as latrines, cesspits, human burials, etc). The *aMPlex Torrent* approach is based on multiplex PCR optimized for highly degraded ancient DNA molecules followed by next generation sequencing of the amplicons on the Ion Torrent platform to efficiently and inexpensively genotype large numbers of archaeological samples [13]. We designed PCR primers to target different taxa of human parasites that are commonly found in archeological sites, taking into account both the short fragment length of ancient DNA molecules and the genetic diversity of the taxa analyzed. Primers were optimized within four multiplex PCRs to allow the identification of 16 species of human tapeworms, roundworms, pinworms and flukes in up to 96 samples simultaneously. The results obtained with this approach on a variety of ca. 100 to 7,200 year-old samples from various archeological and taphonomic contexts were compared with those obtained with the microscopic approach. This study reveals the power of the genetic approach and reports the detection of parasite DNA even in the absence of corresponding eggs. This new approach for the genetic identification of parasites in archeological sites enables researchers to trace back parasite lineages and better understand their evolution. Additionally, the higher resolution analyses possible through genotyping can clarify transmission events from population migrations and contacts between populations, the conquest of new environments, demographic changes and the domestication of animals during the Neolithic, and overall health status of past individuals and populations.

## Materials and Methods

No permits were required for the described study, in particular soil sampling, which complied with all relevant regulations.

### Archeological samples

Sediments from archeological sites have been collected from a wide variety of archeological and taphonomic contexts, geographic areas and periods dating from the Iberian Neolithic (ca. 7,200 BP) to the last century (First World War) (see Table 1).

**Table 1:** List of samples analyzed with results of both genetic (DNA sequences) and microscopic analyses (Eggs).

Sample	Location	Date	Type of sample	Context	DNA sequences	Eggs
**Aa**	Eastern France	20th c. CE	sediment	Barrels (latrine used by soldiers)	*Ascaris*, *Trichuris trichiura*, *Fasciola hepatica*, *Taenia asiatica*	*Ascaris*, *Trichuris*, *Taeniidae*
**Ab**	Eastern France	20th c. CE	sediment	Barrels (latrine used by soldiers)	*Ascaris*, *Trichuris trichiura*, *Fasciola hepatica*	*Ascaris*, *Trichuris*
**B**	Guadeloupe	19th c. CE	sediment	burial	*Trichuris trichiura*	*0*
**Ca**	Northern France	1669–1671	sediment	Latrines	*Dicrocoelium dendriticum*, *Diphyllobothrium*	*Trichuris*
**Cb**	Northern France	1669–1671	sediment	Latrines	*Ascaris*, *Trichuris trichiura*, *Taenia saginata*, *Dicrocoelium dendriticum*, *Fasciola hepatica*	*Ascaris*, *Trichuris*, *Dicrocoelium*, *Fasciola*
**Cc**	Northern France	1669–1671	sediment	Latrines	*Ascaris*, *Trichuris trichiura*, *Taenia solium*, *Dicrocoelium dendriticum*, *Fasciola hepatica*	*Ascaris*, *Trichuris*, *Dicrocoelium*, *Fasciola*
**Da**	Northern France	12-14th c. CE	sediment	Latrines	*Ascaris*, *Trichuris trichiura*, *Taenia solium*, *Enterobius vermicularis*	*Ascaris*, *Trichuris*, *Dicrocoelium*, *Taeniidae*, *Fasciola*
**Db**	Northern France	12-14th c. CE	sediment	Latrines	*Ascaris*, *Trichuris trichiura*, *Fasciola hepatica*, *Enterobius vermicularis*	*Ascaris*, *Trichuris*, *Fasciola*, *Dicrocoelium*
**E**	France	13th c. CE	sediment	Mix of sediments around coxal bones	*Ascaris*, *Dicrocoelium dendriticum*	*Ascaris*, *Trichuris*, *Fasciola*
**F**	Northern France	11-12th c. CE	sediment	Cesspit	*Ascaris*, *Trichuris trichiura*, *Enterobius vermicularis*, *Dicrocoelium dendriticum*, *Fasciola hepatica*	*Ascaris*, *Trichuris*, *Dicrocoelium*, *Fasciola*, *Taeniidae*
**G**	Northern France	8-10th c. CE	coprolite	Ditch	*Fasciola hepatica*	*Ascaris*, *Trichuris*
**H**	Southern France	600–800 CE	sediment	Cesspit	*Ascaris*, *Trichuris trichiura*, *Dicrocoelium dendriticum*, *Fasciola hepatica*, *Enterobius vermicularis*	*Ascaris*, *Trichuris*, *Dicrocoelium*, *Fasciola*
**Ia**	Eastern France	284–476 CE	sediment	Cesspit-latrine	*Ascaris*, *Taenia solium*, *Trichuris trichiura*, *Enterobius vermicularis*	*Ascaris*, *Taeniidae*, *Trichuris*
**Ib**	Eastern France	284–476 CE	sediment	Cesspit-latrine	*Ascaris*, *Trichuris trichiura*	*Fasciola*, *Diphyllobothrium*
**J**	Northern France	150 BCE	sediment	Latrines	*Ascaris*, *Dicrocoelium dendriticum*, *Trichuris trichiura*, *Taenia solium*	*Ascaris*, *Dicrocoelium*, *Fasciola*, *Trichuris*
**Ka**	Iran	2500–1500 BP	sediment	Occupational level	*Ascaris*, *Taenia saginata*, *Dicrocoelium dendriticum*, *Trichuris trichiura*, *Enterobius vermicularis*	*Taeniidae*, *Trichuris*, *Enterobius*
**Kb**	Iran	2500–1500 BP	sediment	Occupational level	*Ascaris*, *Dicrocoelium dendriticum*, *Trichuris trichiura*	*Ascaris*, *Taeniidae*, *Trichuris*
**La**	Northern France	ca 5000 BCE	sediment	Below skeleton in coxal region	*Ascaris*	*0*
**Lb**	Northern France	ca 5000 BCE	sediment	Under skull	*Ascaris*	*0*
**M**	Northern France	6250–5650 BP	sediment	Peripheral ditch	*0*	*Trichuris*, *Taeniidae*, *Dicrocoelium*
**Na**	Spain	7250–7050 BP	sediment	Occupational level	*Ascaris*, *Trichuris trichiura*, *Taenia saginata*, *Dicrocoelium dendriticum*, *Enterobius vermicularis*	*Ascaris*, *Trichuris*, *Taeniidae*
**Nb**	Spain	7250–7050 BP	sediment	Occupational level	*0*	*Ascaris*, *Trichuris*, *Taeniidae*
**Nc**	Spain	7250–7050 BP	sediment	Occupational level	*0*	*Ascaris*, *Trichuris*
**Nd**	Spain	7250–7050 BP	sediment	Occupational level	*0*	*Ascaris*, *Trichuris*, *Taeniidae*
**Ne**	Spain	7250–7050 BP	sediment	Occupational level	*0*	*Ascaris*, *Trichuris*, *Taeniidae*

### Microscopic analysis

Prior to genetic analysis, all samples have been analyzed according to standard paleoparasitological protocols at the laboratory of paleoparasitology of the University of Franche-Comté [14]. For each sample, five grams of sample were rehydrated for one week in a mixed solution of 0.5% tri-sodium phosphate (TSP) and 5% glycerinated water. The samples were then crushed in a mortar and subjected to an ultrasound bath for one minute, before finally being filtered in a column composed of sieves with 315, 160, 50, and 25 μm meshes. Since eggs varied in size between 30–160 μm in length and 15–90 μm in width [15], residues from the two last sieves (50 and 25 μm) were transferred to PVC tubes. Ten slides (22x22 mm) were prepared from each fraction and analyzed under the light microscope, amounting to about 5% of the recovered fraction.

### Extraction and purification of ancient DNA

The pre-PCR procedures were carried out in the high containment ancient DNA analysis facility with positive air pressure of the Jacques Monod Institute using strict experimental procedures as described [16]. Two to ten grams of sediment were ground to fine powder using a Freezer Mill (6750, Spex Certiprep, Metuchen, NJ). The powder was then purified using PowerMax^®^ Soil DNA Isolation Kit (MO BIO Laboratories, Inc. Carlsbad, CA) following the instructions of the manufacturer. Control mock extractions were performed by replacing sediment suspension with 10 mL of distilled water, with one mock extraction for 9 sample extractions. DNA in the 5 mL extract eluted from the MO BIO column was subsequently purified and concentrated on a QIAquick spin column (Qiagen, Hilden, Germany) using 15 mL QG buffer (Qiagen) with 5 mL isopropanol as binding buffer, and PE buffer (Qiagen) for washing. Final elution was performed twice with 45 μL of EB buffer (Qiagen) to which 0.05% Tween-20 was added. To ensure that negative results were not caused by the presence of PCR inhibitors, PCR inhibition of each extract was tested via quantitative PCR (qPCR) on modern genomic DNA, as described [17]. Inhibition in all analyzed samples was found to be low or absent.

### Primer design and testing

We focused on species of parasites common in archeological samples for which modern reference DNA sequences were available. We designed primer pairs to target tapeworms (*Taenia saginata*, *T*. *solium*, *T*. *asiatica*, *Echinococcus granulosus*, *E*. *multilocularis*, *Diphyllobothrium latum*, *D*. *dendriticum*, *D*. *nihonkaiense*), roundworms (*Ascaris lumbricoides*, *A*. *suum*), a whipworm (*Trichuris trichiura*), a pinworm (*Enterobius vermicularis*), and flukes (*Fasciola hepatica*, *F*. *gigantica*, *Dicrocoelium dendriticum*, *D*. *chinensis*). To select for amplicons containing single nucleotide polymorphisms (SNPs) that would allow the identification of human gastro-intestinal helminths in a species-specific manner, we designed primers based on the analysis of 3,352 mitochondrial and nuclear DNA sequences present in GenBank^®^ at the end of 2013. Aligned sequences were used to design primers hybridizing to conserved sequences and flanking one or several diagnostic SNPs for each genus with the exception of *Ascaris lumbricoides* and *A*. *suum*, the distinction of which cannot be achieved using short DNA fragments based on currently available DNA sequences [18,19]. Primer design was performed using the program primer3 within the software Geneious 6.1 [20]. For primer optimization, the software Oligo 6 (Molecular Biology Insights, Inc) was used to select primers with a Td of 65° to 67°C (nearest neighbor method) and for which there was no predicted 3’-dimer having a ΔG below -1.6 kcal/mol. In this way, we designed 82 primer pairs amplifying fragments between 52 and 121 bp and tested them for efficiency and dimer formation using qPCR in a LightCycler 480^®^ (Roche Applied Science, Penzberg, Germany). PCR amplification was carried out at the genomic platform of the Institut Jacques Monod in a 384-well plate using 5 μL of SYBR Green I Master Mix 1x preheated to 37°C and containing 0.5 μM of each primer and 2 μL of DNA, distributed by an epMotion^®^5070 liquid handling robot (Eppendorf, Hamburg, Germany). A cycling program of 8 min of activation at 95°C, followed by 60 cycles at 95°C for 10 sec and 60°C for 1 min was used, with a melting curve established at the end of the run through one melting step (95°C for 5 sec, then 60°C for 1 min and a temperature increase of 0.11°C/sec with continuous fluorescence measurement).

The performance of the primer pairs was tested *in silico* and *in vitro* and primer pairs were required to have an efficiency superior to 90% and not to yield primer-dimers before 40 cycles. Sixteen primer pairs that satisfied these criteria, the two best pairs per each species (S1 Table), were selected. The resulting amplicons were between 52 to 113 bp and included species-specific SNPs (S2 Table).

### Multiplex PCR

We made use of *in silico* prediction for possible dimer formation in multiplex PCRs with the software PriDimerCheck (http://biocompute.bmi.ac.cn/MPprimer/primer_dimer.html). Based on the output of the program, 16 primer pairs were divided into 4 different multiplex reactions to minimize the predicted stability of the possible 3’-annealed dimers. Each multiplex was optimized by testing different MgCl_2_ concentrations (3 mM, 4 mM and 5 mM) and primer concentrations (0.08 μM, 0.15 μM, 0.2 μM, 0.25 μM and 0.3 μM). Each multiplex reaction was mixed in the high containment laboratory of the Institut Jacques Monod using a 4 μL mixture of each targeted DNA at 2.5 pg/μL, in a total volume of 30 μL of 50 mM Tris/HCl pH 8.3 (at 25°C), 10 mM KCl, 5 mM (NH_4_)_2_SO_4_, 3–5 mM MgCl_2_, 1 mg/mL BSA (bovine serum albumin), 0.08–0.3 μM of each primer, 0.25 mM dA/G/CTPs and 0.50 mM dUTP, 2 U of Fast Start DNA polymerase (Roche Applied Science, Penzberg, Germany), and 0.5 U of cod uracil-N-Glycosylase (UNG, Arcticzymes, Tromsø, Norway). MgCl_2_ solution, reaction buffer and BSA were decontaminated by exposing the solutions in UV-pervious tubes (Qubit®, Life Technologies, Carlsbad, CA) to UV light for 10 min at a short distance as described [21]. A negative control with no template was performed for each multiplex reaction, processed in the same way as the samples throughout the whole experimental procedure. The cycling program consisted of 15 min at 37°C (carry-over contamination prevention through digestion by UNG of dUTP-labeled amplicons), 95°C for 10 min (inactivation of UNG and activation of the Fast Start DNA polymerase), followed by 40 cycles at 95°C for 10 sec and 60°C for 1 min and a final extension step at 72°C for 10 min. Cycling was performed in a laboratory of molecular genetics of the Institut Jacques Monod separated from its high containment laboratory.

After the multiplex PCR amplification, simplex qPCR analyses of the amplification products were performed at the genomic platform of the Institut Jacques Monod using each specific primer pair individually, to measure the production of each PCR product and of primer-dimers that might have formed. Each simplex qPCR was pipetted in yet another laboratory of the Institut Jacques Monod, under negative air pressure acting as a DNA trap, as described above using 2 μL of the multiplex product diluted to 1/50^th^. In order to minimize carry-over contamination, the dilution of the PCR product was performed in a dedicated laboratory, physically separated from the high containment laboratory, the modern DNA facility, the post-PCR facility and the genomic platform. These steps established the optimal conditions for each multiplex using qPCR (S3 Table).

When applied to ancient samples, each multiplex PCR was performed using 4 μL of extract using the optimal conditions established as described above and reported in S3 Table. Thus, when five PCR replicates were performed, 20 μL in total (about a quarter of the purified extract) were analyzed with each primer pair.

### Ion torrent library

Ion torrent sequencing adapters were ligated to the replicates of multiplex PCRs in the genomic platform of the Institut Jacques Monod using a Tecan Freedom Evo 100 robot equipped with a 4-channel liquid handling arm using disposable tips, a gripper to move objects, a double thermoblock and an automated vacuum solid phase extraction. First, PCR products were converted to blunt ended fragments with 5’-phosphates using NEBNext^®^ End-Repair Module (New England Biolabs, Ipswich, MA) (1 X NEBNext buffer, 0.1 μL End Repair enzyme mix, 10–30 μL PCR products, for a total volume of 50 μL with an incubation at 25°C for 30 min). Prior to ligation, products were purified with the NucleoSpin 96 PCR clean-up kit (Macherey-Nagel, Düren, Germany) and eluted in 50 μL. Ligation of Ion Torrent barcodes and adapters was performed as follows: 20 μL of purified end-repaired products were incubated with 0.66 μM of barcoded-adapters and 0.66 μM of P1 adapter, 1 X NEBNext ligation buffer and 1 μL of NEBNext Quick ligase for 30 min at 16°C. After the ligation, 60 μL of binding buffer (i.e., NT buffer, Macherey-Nagel) were added and the samples were pooled before purification on a single silica column. The pooled barcoded PCR products were size-selected using the Caliper LabChip XT (Perkin-Elmer, Waltham, MA) to select the PCR products ligated to the adapters. The selected products were then subjected to nick repair and amplification with the OneTaq Hot Start DNA polymerase (NEB) in a 40 μL reaction volume containing Ion Torrent primers A and P1 (0.5 μM), incubated for 20 min at 68°C (nick repair step) and then amplified using the following program: initial denaturation at 94°C for 5 min, (94°C for 15 sec, 60°C for 15 sec, 68°C for 40 sec) for 6 cycles, final elongation at 68°C for 5 min. Products were finally purified using the QIAquick PCR Purification Kit (Qiagen). Size distribution and concentration of the libraries were assessed with an Agilent 2100 Bioanalyzer (Agilent Technologies Inc., Santa Clara, CA). Emulsion PCR and Ion Sphere Particle enrichment were conducted with the Ion OneTouch System™ (Life Technologies) using the Ion OneTouch™200 Template kit v2 DL according to the manufacturer’s protocol. The DNA libraries were sequenced on the Ion Torrent Personal Genome Machine (PGM) Sequencer using the Ion PGM 200 Sequencing Kit and Ion 314 or 316 semiconductor sequencing chips (Life Technologies). It should be noted that the described procedure can be easily adapted to Illumina sequencing technology by using the appropriate adapters if a PGM is not available. The lower sequencing costs of the PGM with Ion 314 chips for up to 200 bp-long sequences, and the superior ability of the PGM to analyze fragments very heterogeneous in size, make it a preferred choice for most applications where the 314 chips provides enough sequencing reads to reliably characterize the PCR products.

### Data analysis

Using the software provided in the Torrent suite on the Ion Torrent server, sequencing reads were demultiplexed and mapped with the Torrent Mapping Alignment Program (TMAP) to a reference fasta sequence created by concatenating the various PCR product reference sequences, each separated by a series of Ns. The sequence corresponding to the primers described herein is provided in S1 File. Following mapping, the.bam and.bai files were grouped in a single directory on a Unix-based computer to be further processed. First, files were renamed to allocate simple descriptive names, using bash commands described in the introduction of the analysis bash script in S4 File. The analysis bash script is dependent on the following programs (and tested with the versions indicated between brackets): featureCounts (Subread package 1.4.6) [22], samtools and bcftools (1.2) [23], and VarScan 2 (2.3.7) [24]. featureCounts uses a.gff file describing the features of the fasta file used for mapping. We provide a.gff file in S3 File corresponding to the PCR products and primers described herein to count the proper PCR products (identified as misc_feature), as well as the reads that contain either the forward (identified as primer_bind) or the reverse (identified as primer_bind_reverse) primers. This should enable the user to detect possible primer dimers, which will have a higher read count for primers than for amplicons. The resulting CountAmplicons.count, CountUpperPrimer.count, and CountLowerPrimer.count files in the count directory report the number of reads obtained for each feature of each sample that was processed by the script, thus allowing identification of the parasite genera detected in each sample. “Samtools mpileup” is used in combination with Varscan to generate a list of the variants detected in each sample. This list is found in the resulting.varscan files in the Varscan directory and allows the identification of parasites at the species level using the information recapitulated in S2 and S3 Tables. Finally, the combination of the “samtools mpileup”, “bcftools call”, and vcfutils.pl vcf2fq is used to generate a consensus sequence from the concatenated PCR products obtained for each sample. The consensus sequences in fasta format found in the Fasta directory within the Consensus directory can be imported into a software environment for multiple alignments to analyze the data in more detail. We used Geneious [20] to import both the consensus fasta and the initial bam files. We performed a multiple sequence alignment of the fasta files in Geneious using MAFFT 7 [25], with manual editing when necessary. The various outputs were verified by analyzing the initial sequencing reads within the bam files displayed in Geneious.

## Results and Discussion

### Principle of the experimental strategy

**Fig 1:**
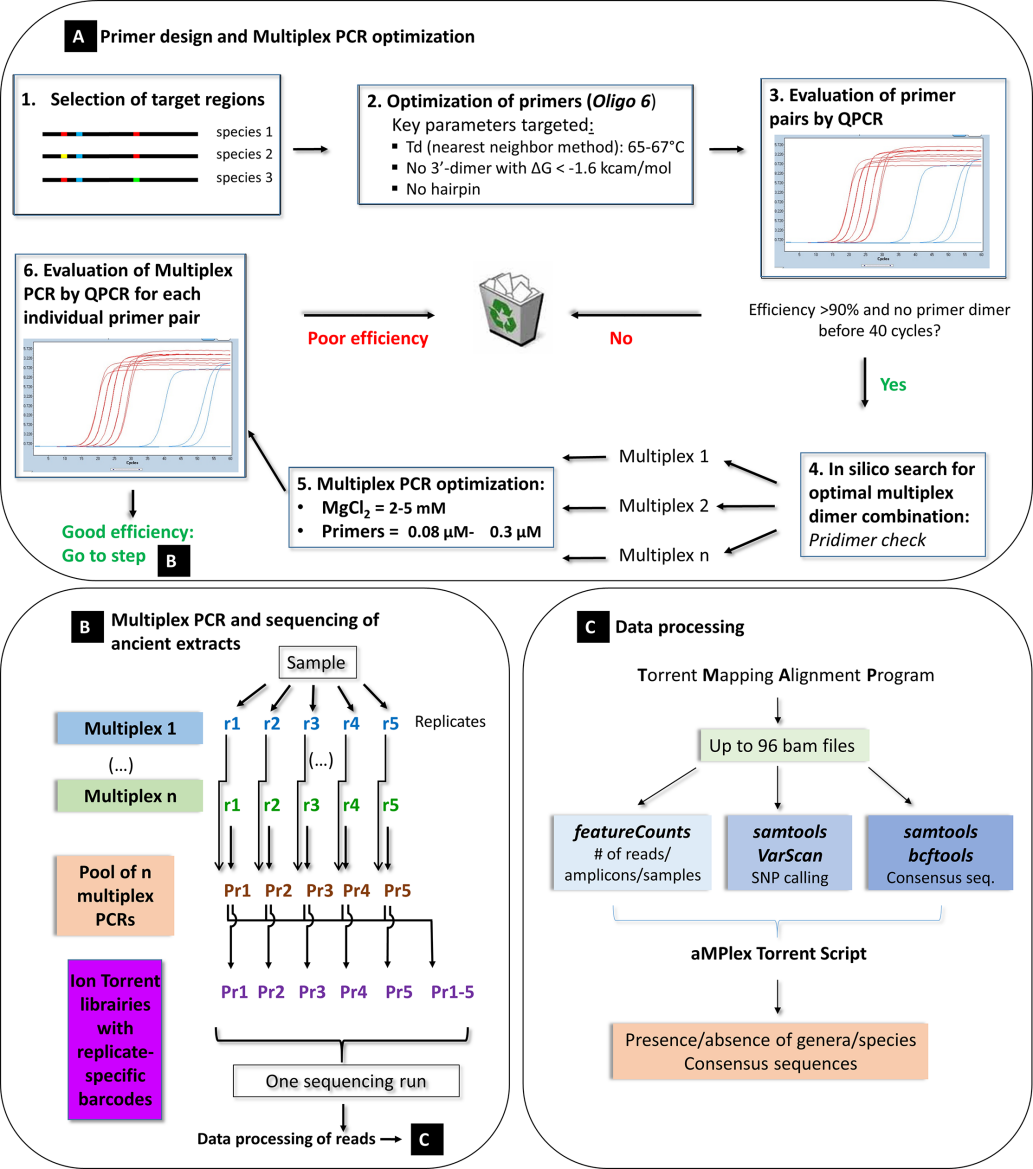
Schematic representation of the workflow of the aMPlex Torrent method for the genotyping of ancient gastrointestinal helminth parasites.

In order to genotype as impartially as possible a large number of more or less well preserved ancient parasite species within a complex DNA mixture containing large quantities of environmental DNA, such as sediments and coprolites, a screening and typing method is needed that satisfies the following criteria: (1) it must take into account the known genetic diversity of the targeted parasites, and (2) it must perform equally well with parasites that are genetically very different. At present, parasites, and in particular gastrointestinal helminths, are best characterized at the level of their mitochondrial DNA. Since mitochondrial DNA also has the highest copy number, the likelihood that rare preserved molecules are detected in an ancient sample is higher than for nuclear DNA. Unfortunately, a large number of parasites cannot be identified at the species level using only a single universal primer pair. This is particularly true when ancient and degraded short DNA molecules are to be analyzed. Thus, primers were designed to fulfill the following conditions: (i) DNA sequences shorter than 80 bp were preferentially targeted, (ii) the primer binding sites must be conserved within a taxonomic order, family or at least genus, and (iii) the DNA sequence between the primers must allow discrimination between species. These constraints argue in favor of an analysis strategy based on a multiplex PCR approach targeting the different branches of the main parasites observed in archeological sites. Therefore, we adapted to the genotyping of ancient gastrointestinal parasites the aMPlex Torrent method which we have recently developed and which combines the sensitivity of the PCR and the efficiency of next-generation sequencing (NGS) [13]. The principle of the “*parasite aMPlex Torrent*” method presented here is described in Fig 1A. Moreover, this method allows the simultaneous analysis of many samples without requiring prior microscopic analysis, which is particularly adapted for archeological projects, and even more so for the analysis of gastrointestinal helminths, which are often preserved in sediments as mixtures of several species.

We designed primer pairs amplifying various short regions of the mitochondrial DNA of the genera *Taenia*, *Echinococcus*, *Diphyllobothrium*, *Trichuris*, *Ascaris*, *Enterobius*, *Fasciola* and *Dicrocoelium* to discriminate 16 species that have been frequently observed in archaeological samples. To increase the reliability of this approach, two diagnostic genomic regions were targeted for each parasite genus. The best two primer pairs were selected for each genus, i.e., those that provide the lowest likelihood of dimer formation and thus the highest sensitivity, and those that performed best in the multiplex PCRs following the strategy described in Fig 1 and in the Materials & Methods section. After ligating sequencing adapters, sequencing was performed on the pooled multiplex PCRs containing sample-specific barcodes on the Ion Torrent sequencing platform [13].

### Performance of the aMPlex Torrent approach for the genotyping of ancient parasites

**Fig 2:**
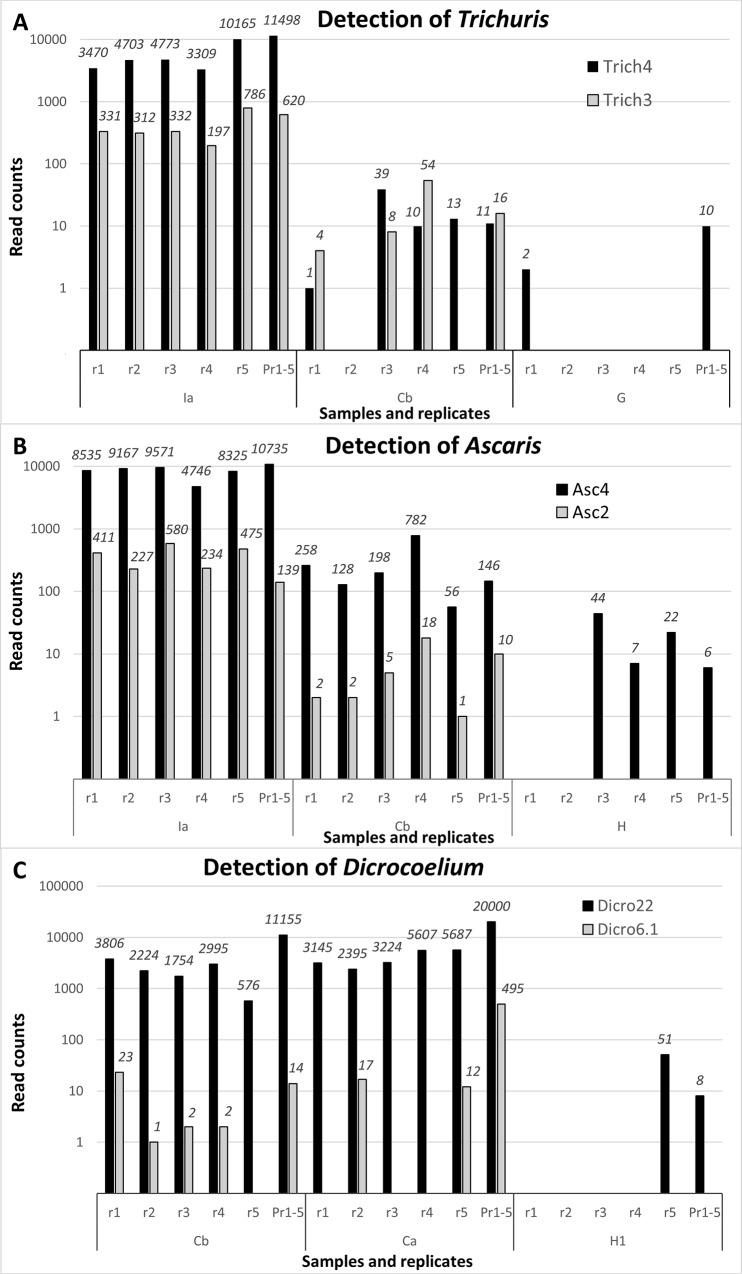
Comparison between the numbers of reads obtained with various primer pairs and samples. Different samples (Ia, Ca, Cb, G, H, H1) in five independent replicates (r1 to r5), as well as in the pool of these five replicates (pool) are represented. **A**. Primer pairs Trich4 and Trich3 that both target *Trichuris trichiura*. **B**. Primer pairs Asc4 and Asc2 that target *Ascaris lumbricoides* and *A*. *suum*. **C**. Primer pairs Dicro22 and Dicro6.1 that target *Dicrocoelium dendriticum and D*. *chinensis*. Black: the more efficient of the two primer pairs, Gray: the less efficient.

The performance of the method was evaluated on a variety of archeological samples from various geographical and cultural origins and taphonomic contexts dating from ca. 100 to 7,200 years ago (Table 1). DNA of each targeted parasite was detected even in samples several thousand years old (see Table 1). This demonstrates that the method is powerful enough to detect rare DNA molecules in the complex DNA mixtures of environmental samples. The reliable detection of rare DNA molecules, however, is not trivial and constitutes a general difficulty in ancient DNA research. Indeed, when analyzing poorly preserved samples, separate PCR assays from the same sample may or may not contain one of the few targeted DNA molecules present in the total extract, rendering reproduction of the results difficult. Moreover, stochastic formation of primer dimers, which can be unpredictable and vary in their appearance over the course of the PCR, can dominate the reaction and reduce sensitivity (S1 Fig). Indeed, if primer-dimers form early during the PCR, lower quantities of target DNA molecules may never be amplified to detectable levels. In the case of multiplex PCR, the situation is even more complex since the probability of heterologous dimer formation is increased exponentially when multiple primer pairs are used simultaneously. As a consequence, the likelihood that DNA molecules are detected in a reliable manner in every PCR is the highest for the most abundant DNA molecules that have been analyzed with the most efficient primer pairs. The rarer the DNA molecules and the less efficient the primer pairs are, the lower the probability will be to detect them reproducibly, as can be seen for all analyzed genera in Fig 2A, 2B and 2C. For each genus, the analysis of three samples containing distinct DNA quantities was deduced from the DNA sequences obtained, ordered according to decreasing numbers of sequences (reads). The samples Ia (Fig 2A and 2B) and Cb (Fig 2C) each provide a high number of reads for each primer pair in almost all replicates (r1-r5). Among the two primer pairs detecting each genus, the more efficient one yielded a higher number of reads (Trich4, Asc4, Dicro22). Other samples contained presumably less DNA (Cb in Fig 2A and 2B; Ca in Fig 2C), yielding a lower number of reads per replicate and not all replicates yielding reads. Accordingly, the less efficient primers (Trich3, Asc2, Dicro6.1) not only yielded fewer reads but these also were found in fewer replicates (e.g., r5 for Cb in Fig 2A and r1, r3 and r4 for Ca in Fig 2C). Samples that contained DNA at the lower limit of detection (G in Fig 2A, H in Fig 2B, H1 in Fig 2C) yielded only few reads in only a subset of replicates and only with the more efficient primer pairs.

In order to achieve maximal sensitivity of parasite DNA detection, it is thus advisable to perform several replicates to identify situations where the DNA quantity may not be sufficient to ensure detection with a single PCR. We consider that three to five replicates are a manageable number for the study of archaeological samples. We compared both strategies, (1) sequencing independently five replicates and (2) pooling these five replicates prior to barcoding for the analysis of a larger number of samples per sequencing run, which reduces the costs of the analysis. For most samples and primer pairs where detection was not achieved in all replicates, the pool of these replicates was able to summarize the result with a single barcode (Pr1-5 in Fig 2). At the extreme lower limit of DNA detectability, i.e., in situations where a small number of reads (<10) was detected in only one out of five replicates, the pool did not always yield a sequence (data not shown). This could be attributed to stochastic fluctuations preventing detection of very low DNA copy numbers that are the result of pooling, which dilutes the sample five-fold. However, because it is difficult to completely exclude contamination issues in this kind of experimental set-up, we recommend refraining from considering samples with such low read counts as positive.

In summary, sequencing costs and sample preparation must be weighed against the potential gain in detection power. Although five independent PCRs provide a more direct estimate of the parasite DNA present in a sample, it is likely that the less expensive pooled PCR approach should be satisfactory for most archaeological situations. The number of reads obtained from each sample may reflect either the initial quantity of parasite DNA and/or its preservation.

### Genotyping of ancient parasites can reveal unknown past genetic diversity

**Fig 3:**
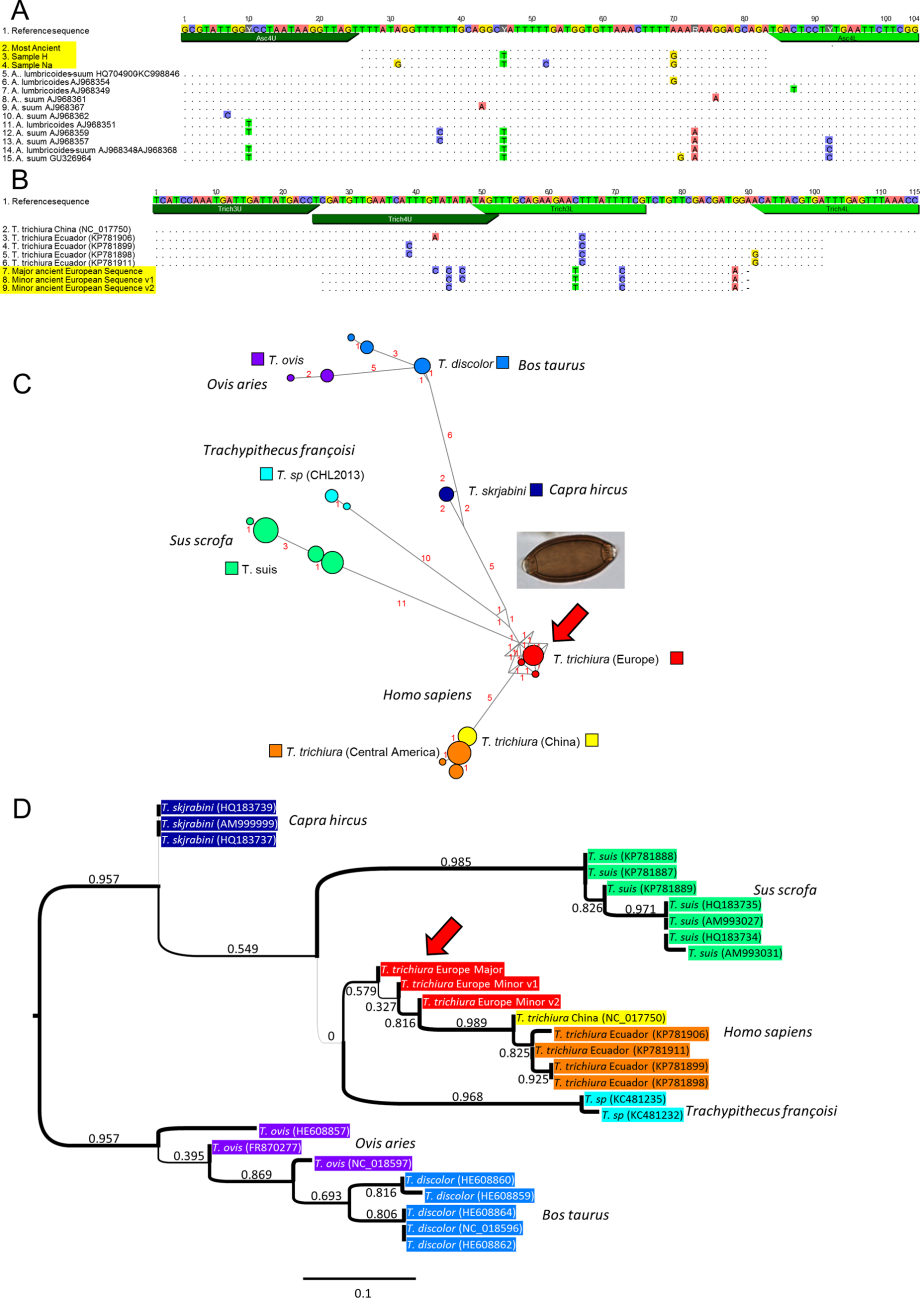
Genotyping reveals past genetic diversity of parasites. **A.** Comparison of ancient and modern genetic diversity of *Ascaris lumbricoides* and *A*. *suum* on the Asc4 PCR fragment. The ancient sequences are highlighted in yellow. When multiple sequences were identical, a single sequence was represented. The variants of the *Ascaris* sequences were found in samples H and Na, as indicated. **B.** Comparison of ancient and modern genetic diversity of *Trichuris trichiura* on the overlapping Trich3 and Trich4 PCR fragments. The variants v1 and v2 of the *Trichuris* sequences were found in samples Da and Ia, respectively. **C.** Median joining network [28] showing the genetic distance separating the human and animal *Trichuris* species, each represented with a different color, and the central position occupied by the sequence of the ancient European human parasite, indicated by a red arrow. The name of the host is indicated in italics. The number of SNPs distinguishing each sequence is indicated in red alongside the corresponding connecting link. The combined sequences, without primers, of the Trich3 and Trich4 fragments were used. **D.** Maximum likelihood (ML) tree showing the phylogenetic relationships between the human and animal *Trichuris* sequences. The tree was constructed using PHYML 3.0 [29] and the Shimodaira-Hasegawa-like branch test (SH) was used to evaluate the statistical support of the nodes, which are indicated along the corresponding branches of the tree. The most distant animal *Trichuris* sequences (*T*. *ovis* & *T*. *discolor*) were used as an outgroup to reveal the relationships between ancient European and modern Asian and American *T*. *trichiura* sequences. The other represented items are as described in panel C.

While the assay was developed to genotype parasite species based on known present-day diversity, the short diagnostic DNA fragments targeted may nevertheless reveal yet undescribed genetic diversity that informs us incidentally on the evolution of these parasites at different time points and geographic locations. Indeed, we could observe in certain samples novel genetic variations within the DNA fragments analyzed. For example, for *Ascaris lumbricoides* and *A*. *suum*, although most positive samples showed a sequence for fragment Asc4 that was identical to the major present-day sequence described, we found in two ancient samples, dated around 7,000 BP, sequences that diverged by two to four SNPs within this 57-nt long fragment (Fig 3A). Also notable, for *Trichuris trichiura*, the sequences that we identified in all European archeological samples were markedly distinct from the currently known genetic diversity, which has been only characterized in China and Central America (Fig 3B). These novel sequences of human-specific *Trichuris trichiura* were clearly distinct from those found in related parasites of domestic animals. Furthermore, the sequences of parasites found in domestic animals diverge markedly in the primer binding sites and thus should be poorly amplified with the primers used (Fig 3C and S2 Fig). Both analysis of *T*. *trichiura* DNA sequences using the median joining network and phylogenetic analysis based on the Maximum Likelihood method reveal that the ancient European DNA sequences are affiliated with the Asian and American DNA sequences and are more basal with respect to those from the domestic animals (Fig 3C and 3D). These preliminary observations raise the possibility that these human parasites may have originated from animals, with the ancient European form being closer to the initial transmission event. More data on the genetic diversity of this parasitic species in the human host is needed to clarify its evolutionary history and better pinpoint the timing and location of possible transmission event(s).

### Comparison between genotyping and microscopic analysis

In order to explore how well a PCR-based approach compares with the traditional microscopic analysis of eggs, we compared the ability to detect seven genera in our complete sample set using both microscopy and genotyping (Table 1). For each genus, both approaches detect identically either the presence or the absence of the corresponding parasite in about three quarters of the cases (Table 2). Overall, the aMPlex Torrent approach generated a taxonomic specificity that could not be achieved with the microscopic identification method. In 14% of the samples parasite DNA was detected in the absence of microscopically detectable eggs. This value raised as high as 20 and 24% in the case of the genera *Dicrocoelium* and *Enterobius*, respectively, suggesting that unidentifiable egg or larval debris can still contain DNA and that morphological and DNA preservation can be uncoupled, presumably influenced by both the properties of the eggs and the taphonomic conditions in the burial site. Notwithstanding the higher taxonomic specificity of the PCR-based approach, DNA was not detected in 15% of our samples despite the presence of identifiable eggs. This result may have been caused by the uneven distribution of eggs in the soil samples analyzed with either method, in particular because in some of the samples microscopic detection relied on the identification of only one or a few eggs per sample. This effect can be minimized with the genotyping approach, where the proportion of extract analyzed in a single PCR is equal to 10 slides analyzed by microscopy. Indeed, the scalability of genotyping can be used to increase the likelihood of detecting rare or less well-preserved material, overcoming this limitation of the reliability of morphology-based methods. Apart from increasing the starting material used for the DNA extracts, performing PCR replicates can ease the screening of a greater proportion of a given sample, i.e., by five times when five PCR replicates are performed. Still, the disparity we observe between microscopic and genetic results may be traced to a combination of the limitations of each method. For microscopy, degraded eggs may show morphological similarities with closely related taxa, and may thus be misidentified or belong to sub-taxa which have yet to be characterized genetically. For genotyping, DNA may remain undetectable by PCR either because the eggs may not contain larvae or DNA might not always be preserved within morphologically identifiable eggs. It should be noted that in this study, even though one of four 7,200 year-old samples (Na in Table 1) yielded sequences for five different species, DNA was not often detected in the samples that were older than 5,000 years. It is well-established that there is a high extent of variability in DNA preservation in old skeletal samples, in particular linked to variations of the burial conditions (e.g., [26]), and it is likely that such a variability in DNA preservation also occurs among parasite eggs. Interestingly, the highest number of the proportion of eggs for which no DNA sequences were obtained was observed for the genus *Trichuris*, followed by *Ascaris* and *Taenia*, suggesting either (i) that a particular characteristic of the eggs of these species may make them less suitable for longer term DNA preservation, (ii) that the larvae may have abandoned the eggs in particular environmental conditions, or (iii) that the eggs belonged to animal-borne parasites containing genetic variability beyond that assessed with the primer pairs used. Thus, for the majority of samples, direct, simultaneous, high-throughput genotyping of soil extracts gave similar results but with a higher level of taxonomic clarification than microscopic identification. Both approaches, however, were found to produce low levels of false negatives for different reasons. To sum up, while for many archeological sites the aMPlex approach alone can produce more specific taxonomic data for many samples with greater efficiency than microscopic egg identification, in order to reduce the rate of false negatives due to the limitations of DNA degradation, this method may be used in tandem with traditional microscopy for samples found to contain severely degraded DNA (see also [27]).

**Table 2:** Comparison of performance of the aMPlex Torrent genotyping and the microscopic approaches to identify parasites in archeological samples up to 7,200 years old.

	DNA/eggs correlated			DNA/eggs uncorrelated		
Genus	No DNA, no eggs	DNA & eggs present	Correlated fraction	DNA, no eggs	Eggs, no DNA	Uncorrelated fraction
*Ascaris*	3	13	64%	4	5	36%
*Trichuris*	2	13	60%	2	8	40%
*Taenia/Echinococcus*	11	5	64%	3	6^*^	36%
*Dicrocoelium*	12	5	68%	5	3	32%
*Fasciola*	13	5	72%	3	4	28%
*Diphyllobothrium*	23	0	92%	1	1	8%
*Enterobius*	18	1	76%	6	0	24%
**Total**	**82**	**42**	**75%**	**24**	**27**	**25%**

The numbers of samples in each category are represented, as well as the proportion of samples, in %, for which the results obtained with both methodologies are correlated or not.

* Microscopic determination cannot distinguish between *Taenia* and *Echinococcus*.

## Conclusions

We adapted the aMPlex Torrent approach [13] to the reliable and efficient detection of 16 different species of gastrointestinal parasites simultaneously in multiple ancient samples, such as sediments from occupational levels and burials of archeological sites, ancient latrines, and coprolites. This is the first time genotyping has been applied to such a wide range of parasites and archaeological sites. These gastrointestinal helminths are the most commonly detected by microscopy in archeological samples [6,7]. Our method allowed for the first time the detection of DNA sequences of ancient *Diphyllobothrium sp*. and *Taenia sp*. While comparative analyses applied to highly degraded material revealed false negatives with both genotyping and microscopy methods, the presented method was powerful enough to detect even highly degraded remains for which no eggs were observed. Thus, we were able to detect *Enterobius vermicularis* in seven samples while up to now its past occurrence has been reported only once in Europe using microscopy, in an ancient German medieval site [10]. We detected *E*. *vermicularis* DNA in samples as old as 7,200 years, indicating that this parasite was already frequent in Western Europe at this time. Moreover, the new genotyping approach allows systematic assignment at the species level for a number of genera, which is rarely possible using microscopy. Finally, it also enables the discovery of ancient genetic diversity not yet described in modern samples. Thus, the aMPlex Torrent method can be used either as a complement to conventional microscopic analyses, or independently to characterize the biodiversity of gastrointestinal parasites in archeological sites. Compared to the microscopy approach, the genotyping approach provides a much more precise identification, improving the investigative power of paleoparasitological studies. The ability to screen many ancient samples for DNA preservation opens the way to future paleogenomic studies that could shed light on the evolution of parasitism, in particular to study the exchange of parasites between humans and animals following animal domestication.

## Supporting Information

S1 Fig Examples of simplex PCR with primer pairs giving rise (b,c) or not (a) to primer dimers. Standard curves performed in duplicates with serial 5-fold dilution of reference DNA are colored in brown, whereas the no template controls (NTC) are represented in red when they give rise to amplification products, and in blue when they do not. a-b) amplification phase of the qPCR, c) melting curve phase of the experiment displayed in panel b. In this latter experiment, four of the six NTCs which give rise to primer dimers have various distinct melting temperatures (Tm). The Tm of the dimer is usually different from that of the product, but it happens sometimes that these values are similar, as shown here for one of the dimers. In such cases, only electrophoretic analyses can distinguish between dimers and PCR products. Three of these dimers were generated at a Ct between 33 and 36, which is similar to the cycles of the PCR where products corresponding to rare initial molecules typical of ancient samples are also detected. A primer pair with such properties is thus not recommended for the detection of ancient DNA molecules. Results obtained with an optimal primer pair have been displayed in panel a) for comparison.
(TIF)

S2 Fig Comparison of the ancient and modern genetic diversity of *Trichuris* species as revealed through the combination of the overlapping Trich3 and Trich4 PCR fragments. The divergence of the primers from sequences of animal species can be seen. Most mismatches between the primers and modern and ancient sequences correspond to G-T mismatch, which are the least destabilizing mismatches (e.g, [1])
1. Pan S, Sun X, Lee JK (2006) DNA stability in the gas versus solution phases: a systematic study of thirty-one duplexes with varying length, sequence, and charge level. J Am Soc Mass Spectrom 17: 1383–1395.
(TIF)

S1 File ref_parasites.fasta (FASTA)

S2 File ref_parasites2.fasta (FASTA)

S3 File ref_parasites.gff (GFF)

S4 File aMPlexmpileup.sh (SH)

S5 File *Ascaris* and *Trichuris* sequences (FASTA)

S6 File All supplementary information in one file. (PDF)

S1 Table Optimal primer pairs targeting gastrointestinal helminths. (DOCX)

S2 Table Sequences of the insert of the PCR products showing the variability found in published sequences within and between species. When several related sequences were available, only the positions differing with the reference sequence are indicated, whereas identical positions are represented by dots. The IUPAC nucleotide ambiguity code was used.
(DOCX)

S3 Table Experimental conditions for the various multiplex PCRs. Optimal MgCl_2_, and primer concentrations used for each individual multiplex PCR are shown.
(DOCX)

S4 Table Location of the diagnostic SNPs for species discrimination to interpret the varscan data files when using the ref_parasites fasta file for mapping the reads. (DOCX)
