# Supplementary figures and images for: A New High-Throughput Approach to Genotype Ancient Human Gastrointestinal Parasites
Source: PLoS One. 2016 Jan 11;11(1):e0146230. doi: 10.1371/journal.pone.0146230 (PMC4709038; doi:10.1371/journal.pone.0146230)

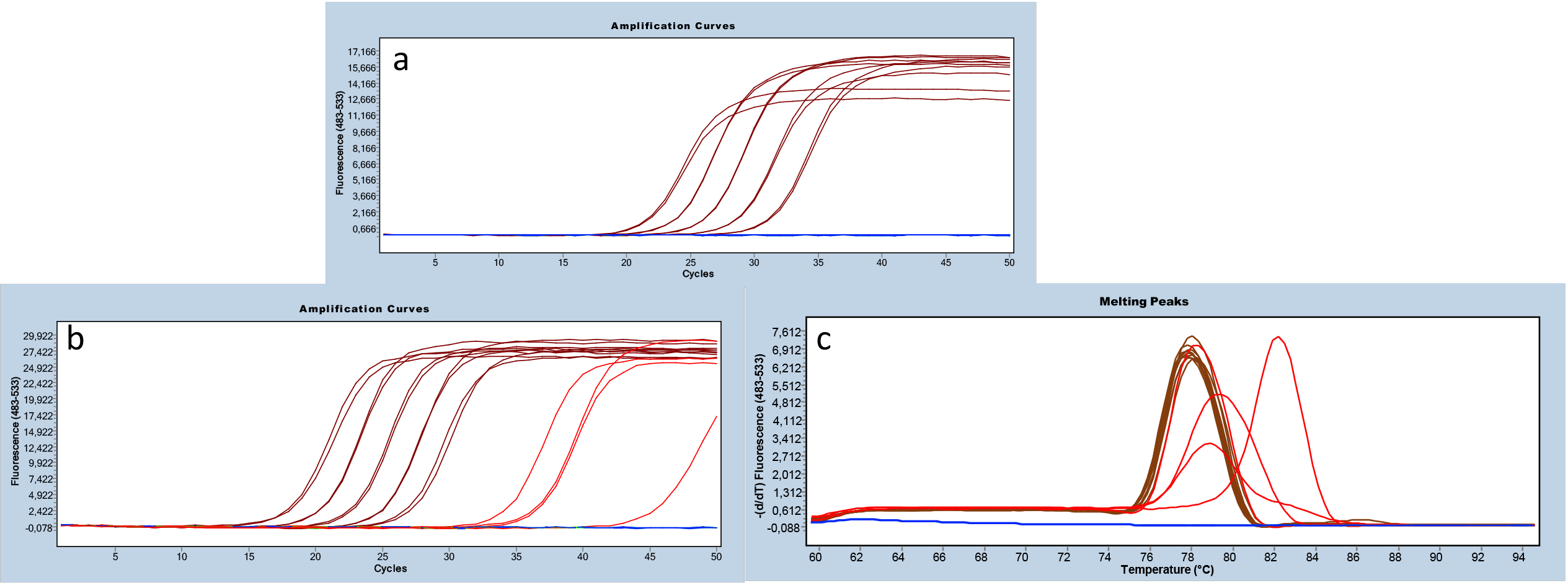

Supplement: S1 Fig — Standard curves performed in duplicates with serial 5-fold dilution of reference DNA are colored in brown, whereas the no template controls (NTC) are represented in red when they give rise to amplification products, and in blue when they do not. a-b) amplification phase of the qPCR, c) melting curve phase of the experiment displayed in panel b. In this latter experiment, four of the six NTCs which give rise to primer dimers have various distinct melting temperatures (Tm). The Tm of the dimer is usually different from that of the product, but it happens sometimes that these values are similar, as shown here for one of the dimers. In such cases, only electrophoretic analyses can distinguish between dimers and PCR products. Three of these dimers were generated at a Ct between 33 and 36, which is similar to the cycles of the PCR where products corresponding to rare initial molecules typical of ancient samples are also detected. A primer pair with such properties is thus not recommended for the detection of ancient DNA molecules. Results obtained with an optimal primer pair have been displayed in panel a) for comparison. (TIF) [file pone.0146230.s001.tif]

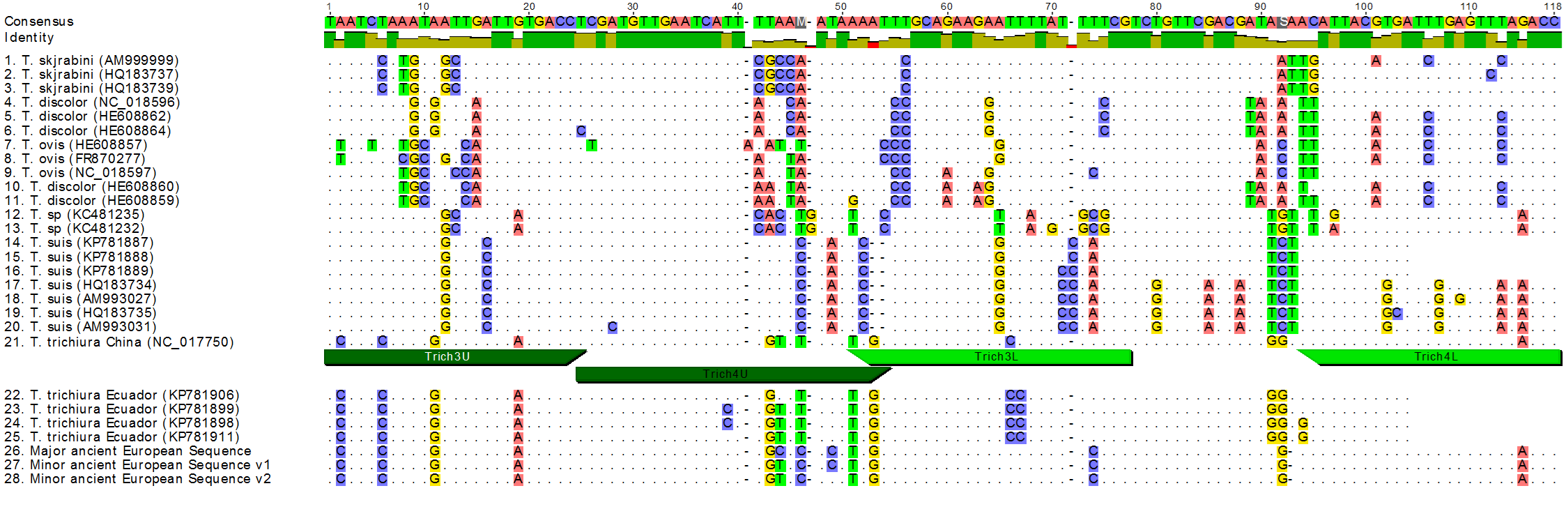

Supplement: S2 Fig — The divergence of the primers from sequences of animal species can be seen. Most mismatches between the primers and modern and ancient sequences correspond to G-T mismatch, which are the least destabilizing mismatches (e.g, [1]) 1. Pan S, Sun X, Lee JK (2006) DNA stability in the gas versus solution phases: a systematic study of thirty-one duplexes with varying length, sequence, and charge level. J Am Soc Mass Spectrom 17: 1383–1395. (TIF) [file pone.0146230.s002.tif]
