# Supplementary material for: A New High-Throughput Approach to Genotype Ancient Human Gastrointestinal Parasites
Source: PLoS One. 2016 Jan 11;11(1):e0146230. doi: 10.1371/journal.pone.0146230 (PMC4709038; doi:10.1371/journal.pone.0146230)
Supplement: S6 File — (PDF) [file pone.0146230.s008.pdf]

Supplementary data for:

A new high-throughput approach to genotype ancient human gastrointestinal parasites

Nathalie M. L. Côté, Julien Daligault, Mélanie Pruvost, E. Andrew Bennett, Olivier Gorgé, Silvia Guimaraes, , Nicolas Capelli, Matthieu Le Bailly, Eva-Maria Geigl*, Thierry Grange*

About the Authors

N. M. L. Côté, J. Daligault, M. Pruvost, O. Gorgé, S. Guimaraes, E. A. Bennett E.-M. Geigl, T. Grange

Institut Jacques Monod, CNRS, University Paris Diderot, UMR 7592, Epigenome and Paleogenome group, 15 rue Hélène Brion, 75205 Paris cedex 13, France.

N. M. L. Côté, M. Le Bailly, N. Capelli

University of Bourgogne Franche-Comte, CNRS UMR 6249 Chrono-environment, 16 route de Gray, 25030, Besançon cedex, France.

*equal contribution

Corresponding Authors

eva-maria.geigl@ijm.fr; thierry.grange@ijm.fr

Primer name	*Organism* Gene	Size of amplicon (bp)	Primer sequences
Tae23	*Taenia*<br>Cytochrome oxydase 1 (cox1)	73	U : GAG GTT TTA GGT TCW TAT GGT T<br>L : CAT TAT GAG AAG CYA CAG GAC T
Tae32	*Taenia*<br>Cytochrome b (cytb)	113	U : ATA TGG CTC GTG CTT TGT ATT ATT C<br>L: AGG TAA TAT ATA TCC WGT AAA AGC CTC
Echino5	*Echinococcus*<br>Phosphoenolpyruvate carboxykinase (pepck, nuclear)	109	U : CAC ATG TTG CTG AAG GCG TTA<br>L : AAT GTA CGG TCG TTC ATG CAG
Echino23	*Echinococcus*<br>cox1	99	U: TTT TGA TCC GTT AGG TGG T<br>L: CCA AAT CCA GGC ARA ATC
Diphyllo2	*Diphyllobothrium*<br>cox1	52	U: GTT GTG TGG GGG CAT CAT A<br>L:CAG CCG TCT TTA CAT CTA AAC C
Diphyllo23	*Diphyllobothrium*<br>cox1	67	U: TTT AYG GGT TGT TAT TTG CT<br>L: ATA TGA TGC CCC CAC ACA
Asc2	*Ascaris*<br>cytb	74	U: GCC AAA GCA CCA TCA TTA GAA TA<br>L : GGT ATG GTT TTG GGT TTT CAG A
Asc4	*Ascaris*<br>NADH dehydrogenase 1 (nad1)	104	U : GCG TAT TGG YCC TAA TAA GGT TAG T<br>L :CCG AAG AAT TCA RAG GAG TCA
Trich3	*Trichuris trichiura*<br>Large ribosomal subunit (LSU)	74	U :TCA TCC AAA TGA TTG ATT ATG ACC T<br>L :CGA AAA TAA AGT TCT TCT GCA AAC TA
Trich4	*Trichuris trichiura*<br>LSU	91	U: TCG ATG TTG AAT CAT TTG TAT ATA TAG T<br>L:GGT TTA AAC TCA AAT CAC GTA ATG T
Dicro22	*Dicrocoelium*<br>Internal transcribed spacer 2 (nuclear)	76	U : TAC ACA CAC CTA GTT ATC AGA CAG<br>L: ACA GAC CGC GCA TAA ATA
Dicro6.1	*Dicrocoelium*<br>nad1	77	U: TAA GTA TAA GTT TST GGT TTC TCA GTT TC<br>L: AAG CWA CCA AAA TCA TCA AAA ACA
Fas2	*Fasciola*<br>cox1	69	U : GTT GAT TGG GGG KTT TGG TA<br>L : CGA GGC AAA TTC AAA TCA GG
Fas3	*Fasciola*<br>rRNA18s (nuclear)	85	U : AAC CTG CGG AAG GAT CAT TA<br>L: GCA AAT TTT TAT CGC ATG ACA
Entero2	*Enterobius vermicularis*<br>SL1RNA (nuclear)	56	U: TTT ATT TCC AAG CCA CAG ACT CA<br>L: AAT TTC TCG TTC CGG CTC AG
Entero4	*Enterobius vermicularis*<br>cox1	53	U: CTG TGC CRA CTG GGG TAA AG<br>L : TCC CCC TAT CAA AGT CAA CAA C

S1 Table. Optimal primer pairs targeting gastrointestinal helminths.

Genus	species	PCR product 1	PCR product 2
Taenia	*solium*	**Tae23**<br>**CA**T**C**TAG**C**TGTT**TG**GT**GA**ATTTTTTTATG<br>T............................<br>.................G...........	**Tae32**<br>AAGT**T**A**T**A**G**AAAGAAGGGTGT**G**TGAAATGT**T**GG**G**TTT**A**TTTT**A**TATTTATT**GG**TTATGGG**A**G<br>..C.........<br>.................................A..................G.........
	saginata	.G.....T....AT..RG...........<br>.G.A...T....AT...G...........	........A............A........A......G....G........A........T.
	asiatica	.......T....AT...G...........	........A............A........A......G....G........A........T.
Echino-coccus	*granulosus*	**Echino5**<br>AAGGCATCCTTGGCCAATGGATCTCGCCAAAAGATCTTGACGAATCCCTTAGGGATCGCTTCCCTGG	**Echino23**<br>GGTGATCCTATTTT**G**TTTCAACATATGTT**T**TGGTTTTTTGG**C**CATCC**T**GAGGTTTATGTG**T**T<br>..............A................................H..............<br>..............A..............Y...........T.....T..............<br>..............A...C.
	multilo-cularis	....	............Y.A..........................T.....G...........T..<br>..............R..........................T.....G...........G..
Diphyllo-bothrium	*latum*	**Diphyllo2**<br>TGTTTAC**G**GT**G**	**Diphyllo23**<br>ATGTT**T**TCTATAGT**T**TG**T**TT**A**GGTAG**A**GT
	dendri-ticum	.......**A**..**A**	.....Y........C..C..G.....T..
	nihon-kaiense	.......**A**...	.................C........T..
Ascaris	*lumbri-coides and suum*	**Asc2**<br>TTTTGACTGGTA**CT**TTTTTG**G**CTTTTTAT<br>............**GT**......**R**........<br>............**AC**..... **G**........	**Asc4**<br>TTTATAGGTTT**T**TTGCAGGC**C**ATTTTTGATGGTGTTAAACTTTTAAA**G**AA**R**GAGCAGA<br>...........**Y**........**T**..........................**A**..**G**.......
Trichuris	*trichiura*	**Trich3**<br>CGATGTTGAATCATTTGTATATA	**Trich4**<br>TTGCAGAAGAACTTTATTTTCGTCTGTTCGACGATGGA
Dicro-coelium	*dendri-ticum*	**Dicro22**<br>GTGGAGATGTGTCTACGGAGTCGTGGCT	**Dicro6.**1<br>GGGATTGGTTGTCTTG**G**GTTGGTT
	chinensis	....	................**A**.......

Fasciola	*hepatica*	**Fas2**<br>`ATTATTTGTTGCC`**`YT`**`TATTGTTAGG`**`T`**`ATT`<br>`.............CY..........G...`	**Fas3**<br>`CCTGAAAATCTACTCT`**`C`**`ACACAAGCGATACACGTGTGACCGTCA`
	gigantica	`.Y.....A.....T..GC.T..G......`<br>`.............T..GC.T..G......`<br>`.......A..A..T..GC.T.........`	`................`**`T`**`...........................`
Entero-bius	*vermicu-laris*	**Entero2**<br>`CTGATGTTCATGT`	**Entero4**<br>`GTTTTTAGTTG`

S2 Table. Sequences of the insert of the PCR products showing the variability found in published sequences within and between species. When several related sequences were available, only the positions differing with the reference sequence are indicated, whereas identical positions are represented by dots. The IUPAC nucleotide ambiguity code was used.

**Multiplex 1** 4 mM $MgCl_2$		**Multiplex 2** 5 mM $MgCl_2$		**Multiplex 3** 4mM $MgCl_2$		**Multiplex 4** 5 mM $MgCl_2$	
primers	[µM]	primers	[µM]	primers	[µM]	primers	[µM]
Tae32	0.15	Tae23	0.2	Trich3	0.25	Dicro6.1	0.2
Echino5	0.08	Trich4	0.08	Entero4	0.08	Asc4	0.2
Diphyllo2	0.08	Dicro22	0.08			Fas3	0.08
		Entero2	0.08			Fas2	0.08
		Diphyllo23	0.08			Echino23	0.08
						Asc2	0.08

S3 Table. Experimental conditions for the various multiplex PCRs. Optimal $MgCl_2$, and primer concentrations used for each individual multiplex PCR are shown.

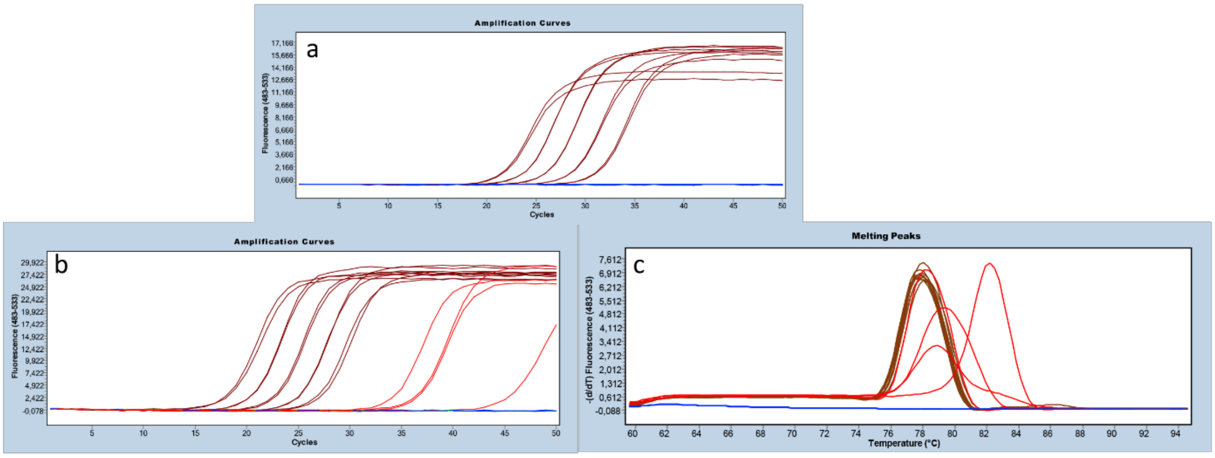

S1 Fig. Examples of simplex PCR with primer pairs giving rise (b,c) or not (a) to primer dimers. Standard curves performed in duplicates with serial 5-fold dilution of reference DNA are colored in brown, whereas the no template controls (NTC) are represented in red when they give rise to amplification products, and in blue when they do not. a-b) amplification phase of the qPCR, c) melting curve phase of the experiment displayed in panel b. In this latter experiment, four of the six NTCs which give rise to primer dimers have various distinct melting temperatures (Tm). The Tm of the dimer is usually different from that of the product, but it happens sometimes that these values are similar, as shown here for one of the dimers. In such cases, only electrophoretic analyses can distinguish between dimers and PCR products. Three of these dimers were generated at a Ct between 33 and 36, which is similar to the cycles of the PCR where products corresponding to rare initial molecules typical of ancient samples are also detected. A primer pair with such properties is thus not recommended for the detection of ancient DNA molecules. Results obtained with an optimal primer pair have been displayed in panel a) for comparison.

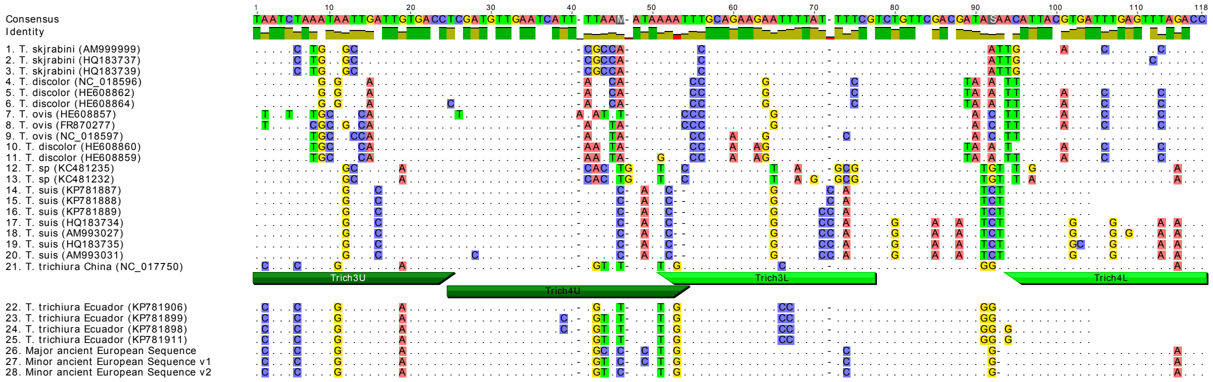

S2 Fig. Comparison of the ancient and modern genetic diversity of *Trichuris* species as revealed through the combination of the overlapping Trich3 and Trich4 PCR fragments. The divergence of the primers from sequences of animal species can be seen. Most mismatches between the primers and modern and ancient sequences correspond to G-T mismatch, which are the least destabilizing mismatches (e.g., [1]).

1. Pan S, Sun X, Lee JK (2006) DNA stability in the gas versus solution phases: a systematic study of thirty-one duplexes with varying length, sequence, and charge level. J Am Soc Mass Spectrom 17: 1383-1395.

Files for bioinformatics analyses of the data

1. Fasta file for mapping and downstream SNP calling analyses

S1 File. ref_parasites.fasta

```
# To be used with the following script, name this file ref_parasites.fasta
>ref_parasites
NNNNNNNNNNNNNNNNNNNNNNNNNNNNNNNNNNNNNNNNNNNNNNNNNNGAGGTTTTAGGTTCWTATGGTTYRTMTAGY
TGTTWKGTGRATTTTTTTATGAGTCCTGTRGCTTCACATAATGNNNNNNNNNNNNNNNNNNNNNNNNNNNNNNNNNNNNN
NNNNNNNNNNNNNATATGGCTCGTGCTTTGTATTATTCAAGTTAWARAAAGAAGGGTGTRTGAAATGTWGGRTTTRTTTT
RTATTTATTRSTTATGGGWGAGGCTTTTACWGGATATATATTACCTNNNNNNNNNNNNNNNNNNNNNNNNNNNNNNNNNN
NNNNNNNNNNNNNNNNCACATGTTGCTGAAGGCGTTAAAGGCATCCTTGGCCAATGGATCTCGCCAAAAGATCTTGACGA
ATCCCTTAGGGATCGCTTCCCTGGCTGCATGAACGACCGTACATTNNNNNNNNNNNNNNNNNNNNNNNNNNNNNNNNNNN
NNNNNNNNNNNNNNNNTTTGATCCGTTAGGTGGTGGTGATCCTATTYTRTTTCARCATATGTTYTGGTTTTTTGGYCATC
CNGAGGTTTATGTKYTGATTYTGCCTGGATTTGGNNNNNNNNNNNNNNNNNNNNNNNNNNNNNNNNNNNNNNNNNNNNNN
NNNNGTTGTGTGGGGGCATCATATGTTTACRGTRGGTTTAGATGTAAAGACGGCTGNNNNNNNNNNNNNNNNNNNNNNNN
NNNNNNNNNNNNNNNNNNNNNNNNNNTTTAYGGGTTGTTATTTGCTATGTTTTCTATAGTYTGYTTRGGTAGWGTTGTGT
GGGGGCATCATATNNNNNNNNNNNNNNNNNNNNNNNNNNNNNNNNNNNNNNNNNNNNNNNNNNNGCCAAAGCACCATCATT
AGAATAATAAAAAGYCAAAAARBTACCAGTCAAAATCTGAAAACCCAAAACCATACCNNNNNNNNNNNNNNNNNNNNNNN
NNNNNNNNNNNNNNNNNNNNNNNNNNNGCGTATTGGYCCTAATAAGGTTAGTTTTATAGGTTTYTTGCAGGCYATTTTTG
ATGGTGTTAAACTTTTRRARAAGGAGCAGATGACTCCTYTGAATTCTTCGGNNNNNNNNNNNNNNNNNNNNNNNNNNNNN
NNNNNNNNNNNNNNNNNNNNNTCATCCAAATGATTGATTATGACCTCGATGTTGAATCAYTTGWAYAYATAGTTTGCAGA
AGAACTTTATTTCGNNNNNNNNNNNNNNNNNNNNNNNNNNNNNNNNNNNNNNNNNNNNNNNNNNNTCGATGTTGAATCAT
TTGTATATATAGTTTGCAGAAGAACYTTATTTTCGTCTGTTCGACGATGGAACATTACGTGATTTGAGTTTAAACCNNNN
NNNNNNNNNNNNNNNNNNNNNNNNNNNNNNNNNNNNNNNNNNNNNNTACACACACCTAGTTATCAGACAGGTGGAGATGT
GTCTACGGAGTCGTGGCTCAGTAMTATTTATGCGCGCTCTGTNNNNNNNNNNNNNNNNNNNNNNNNNNNNNNNNNNNNNN
NNNNNNNNNNNNTAAGTATAAGTTTSTGGTTTCTCAGTTTCGGGATTGGTTGTCTTGRGTTGGTTTGTTTTTGATGATTT
TGGTWGCTTNNNNNNNNNNNNNNNNNNNNNNNNNNNNNNNNNNNNNNNNNNNNNNNNNNGTTGATTGGGGGKTTTGGTAA
TTATTTRTTGCCYTTRYTKTTGGGKATTCCTGATTTGGATTTGCCTCGNNNNNNNNNNNNNNNNNNNNNNNNNNNNNNNN
NNNNNNNNNNNNNNNNNNAACCTGCGGAAGGATCATTACCTGAAAATCTACTCTYACACAAGCGATACACGTGTGACCGT
CATGTCATGCGATAAAAATTTGCNNNNNNNNNNNNNNNNNNNNNNNNNNNNNNNNNNNNNNNNNNNNNNNNNNTTTATTT
CCAAGCCACAGACTCACTGATGTTCATGTCTGAGCCGGAACGAGAAATTNNNNNNNNNNNNNNNNNNNNNNNNNNNNNNN
NNNNNNNNNNNNNNNNNNNCTGTGCCRACTGGGGTTAAGGTYTTYAGTTGGTTGTTGACTTTGATAGGGGGTNNNNNNNN
NNNNNNNNNNNNNNNNNNNNNNNNNNNNNNNNNNNNNNNNNN
```

2. Fasta file for downstream consensus sequence generations

S2 File. ref_parasites2.fasta

```
# To be used with the following script, name this file ref_parasites2.fasta
>ref_parasites
NNNNNNNNNNNNNNNNNNNNNNNNNNNNNNNNNNNNNNNNNNNNNNNNNNGAGGTTTTAGGTTCWTATGGTTCATCTAGC
TGTTTGGTGAATTTTTTTATGAGTCCTGTRGCTTCACATAATGNNNNNNNNNNNNNNNNNNNNNNNNNNNNNNNNNNNNN
NNNNNNNNNNNNNATATGGCTCGTGCTTTGTATTATTCAAGTTATAGAAAGAAGGGTGTGTGAAATGTTGGGTTTATTTT
ATATTTATTGCTTATGGGAGAGGCTTTTACWGGATATATATTACCTNNNNNNNNNNNNNNNNNNNNNNNNNNNNNNNNNN
NNNNNNNNNNNNNNNNCACATGTTGCTGAAGGCGTTAAAGGCATCCTTGGCCAATGGATCTCGCCAAAAGATCTTGACGA
ATCCCTTAGGGATCGCTTCCCTGGCTGCATGAACGACCGTACATTNNNNNNNNNNNNNNNNNNNNNNNNNNNNNNNNNNN
NNNNNNNNNNNNNNNNTTTGATCCGTTAGGTGGTGGTGATCCTATTTTATTTCAACATATGTTTTGGTTTTTTGGCCATC
CTGAGGTTTATGTGTTGATTYTGCCTGGATTTGGNNNNNNNNNNNNNNNNNNNNNNNNNNNNNNNNNNNNNNNNNNNNNN
NNNNGTTGTGTGGGGGCATCATATGTTTACAGTAGGTTTAGATGTAAAGACGGCTGNNNNNNNNNNNNNNNNNNNNNNNN
NNNNNNNNNNNNNNNNNNNNNNNNNNTTTAYGGGTTGTTATTTGCTATGTTTTCTATAGTCTGCTTGGGTAGTGTTGTGT
GGGGGCATCATATNNNNNNNNNNNNNNNNNNNNNNNNNNNNNNNNNNNNNNNNNNNNNNNNNNNGCCAAAGCACCATCATT
AGAATAATAAAAAGCCAAAAAAGTACCAGTCAAAATCTGAAAACCCAAAACCATACCNNNNNNNNNNNNNNNNNNNNNNN
NNNNNNNNNNNNNNNNNNNNNNNNNNNGCGTATTGGYCCTAATAAGGTTAGTTTTATAGGTTTTTTGCAGGCTATTTTTG
ATGGTGTTAAACTTTTAAAAAAGGAGCAGATGACTCCTYTGAATTCTTCGGNNNNNNNNNNNNNNNNNNNNNNNNNNNNN
NNNNNNNNNNNNNNNNNNNNNTCATCCAAATGATTGATTATGACCTCGATGTTGAATCATTTGTATATATAGTTTGCAGA
AGAACTTTATTTCGNNNNNNNNNNNNNNNNNNNNNNNNNNNNNNNNNNNNNNNNNNNNNNNNNNNTCGATGTTGAATCAT
TTGTATATATAGTTTGCAGAAGAACCTTATTTTCGTCTGTTCGACGATGGAACATTACGTGATTTGAGTTTAAACCNNNN
NNNNNNNNNNNNNNNNNNNNNNNNNNNNNNNNNNNNNNNNNNNNNNTACACACACCTAGTTATCAGACAGGTGGAGATGT
GTCTACGGAGTCGTGGCTCAGTAATATTTATGCGCGCTCTGTNNNNNNNNNNNNNNNNNNNNNNNNNNNNNNNNNNNNNN
NNNNNNNNNNNNTAAGTATAAGTTTSTGGTTTCTCAGTTTCGGGATTGGTTGTCTTGGGTTGGTTTGTTTTTGATGATTT
TGGTWGCTTNNNNNNNNNNNNNNNNNNNNNNNNNNNNNNNNNNNNNNNNNNNNNNNNNNGTTGATTGGGGGKTTTGGTAA
TTATTTGTTGCCCTTATTGTTAGGGATTCCTGATTTGGATTTGCCTCGNNNNNNNNNNNNNNNNNNNNNNNNNNNNNNNN
NNNNNNNNNNNNNNNNNNAACCTGCGGAAGGATCATTACCTGAAAATCTACTCTCACACAAGCGATACACGTGTGACCGT
CATGTCATGCGATAAAAATTTGCNNNNNNNNNNNNNNNNNNNNNNNNNNNNNNNNNNNNNNNNNNNNNNNNNNTTTATTT
CCAAGCCACAGACTCACTGATGTTCATGTCTGAGCCGGAACGAGAAATTNNNNNNNNNNNNNNNNNNNNNNNNNNNNNNN
NNNNNNNNNNNNNNNNNNNCTGTGCCRACTGGGGTTAAGGTTTTTAGTTGGTTGTTGACTTTGATAGGGGGTNNNNNNNN
```

NNNNNNNNNNNNNNNNNNNNNNNNNNNNNNNNNNNNNNNNNNNNNNN

3. gff file for mapping and downstream analyses

S3 File. ref_parasites.gff

```
##gff-version 3
##source-version geneious 6.1.8
ref_parasitesGeneious	misc_feature	85	89	.	.	.	gene_id "Taenia23";
ref_parasitesGeneious	misc_feature	229	232	.	.	.	gene_id "Taenia32";
ref_parasitesGeneious	misc_feature	385	395	.	.	.	gene_id "Echinococcus5";
ref_parasitesGeneious	misc_feature	538	546	.	.	.	gene_id "Echinococcus23";
ref_parasitesGeneious	misc_feature	668	672	.	.	.	gene_id "Diphyllobothrium2";
ref_parasitesGeneious	misc_feature	782	784	.	.	.	gene_id "Diphyllobothrium23";
ref_parasitesGeneious	misc_feature	898	902	.	.	.	gene_id "Ascaris2";
ref_parasitesGeneious	misc_feature	1036	1044	.	.	.	gene_id "Ascaris4";
ref_parasitesGeneious	misc_feature	1185	1187	.	.	.	gene_id "Trichuris3";
ref_parasitesGeneious	misc_feature	1320	1328	.	.	.	gene_id "Trichuris4";
ref_parasitesGeneious	misc_feature	1450	1452	.	.	.	gene_id "Dicrocoelium22";
ref_parasitesGeneious	misc_feature	1571	185	.	.	.	gene_id "Dicrocoelium61";
ref_parasitesGeneious	misc_feature	1684	1704	.	.	.	gene_id "Fasciola2";
ref_parasitesGeneious	misc_feature	1809	1813	.	.	.	gene_id "Fasciola3";
ref_parasitesGeneious	misc_feature	1937	1938	.	.	.	gene_id "Enterobius2";
ref_parasitesGeneious	misc_feature	2044	2047	.	.	.	gene_id "Enterobius4"
ref_parasitesGeneious	primer_bind	51	72	.	+	.	gene_id "Tae23U";
ref_parasitesGeneious	primer_bind	174	198	.	+	.	gene_id "Tae32U";
ref_parasitesGeneious	primer_bind	337	357	.	+	.	gene_id "Echino5U";
ref_parasitesGeneious	primer_bind	496	514	.	+	.	gene_id "Echino23U";
ref_parasitesGeneious	primer_bind	645	663	.	+	.	gene_id "Diphyllo2U";
ref_parasitesGeneious	primer_bind	747	766	.	+	.	gene_id "Diphyllo23U";
ref_parasitesGeneious	primer_bind	864	236	.	+	.	gene_id "Asc2U";
ref_parasitesGeneious	primer_bind	988	1012	.	+	.	gene_id "Asc4U";
ref_parasitesGeneious	primer_bind	1142	1166	.	+	.	gene_id "Trich3U";
ref_parasitesGeneious	primer_bind	1266	1292	.	+	.	gene_id "Trich4U";
ref_parasitesGeneious	primer_bind	1407	1430	.	+	.	gene_id "Dicro22U";
ref_parasitesGeneious	primer_bind	1533	1561	.	+	.	gene_id "Dicro61U";
ref_parasitesGeneious	primer_bind	1660	1679	.	+	.	gene_id "Fas2U";
ref_parasitesGeneious	primer_bind	1779	1798	.	+	.	gene_id "Fas3U";
ref_parasitesGeneious	primer_bind	1914	1936	.	+	.	gene_id "Entero2U";
ref_parasitesGeneious	primer_bind	2020	2039	.	+	.	gene_id "Entero4U";
ref_parasitesGeneious	primer_bind_reverse	102	123	.	-	.	gene_id "Tae23L";
ref_parasitesGeneious	primer_bind_reverse	261	286	.	-	.	gene_id "Tae32L";
ref_parasitesGeneious	primer_bind_reverse	425	445	.	-	.	gene_id "Echino5L";
ref_parasitesGeneious	primer_bind_reverse	577	594	.	-	.	gene_id "Echino23L";
ref_parasitesGeneious	primer_bind_reverse	675	696	.	-	.	gene_id "Diphyllo2L";
ref_parasitesGeneious	primer_bind_reverse	796	813	.	-	.	gene_id "Diphyllo23L";
ref_parasitesGeneious	primer_bind_reverse	915	937	.	-	.	gene_id "Asc2L";
ref_parasitesGeneious	primer_bind_reverse	1071	1091	.	-	.	gene_id "Asc4L";
ref_parasitesGeneious	primer_bind_reverse	1190	1215	.	-	.	gene_id "Trich3L";
ref_parasitesGeneious	primer_bind_reverse	1332	1356	.	-	.	gene_id "Trich4L";
ref_parasitesGeneious	primer_bind_reverse	1459	1482	.	-	.	gene_id "Dicro22L";
ref_parasitesGeneious	primer_bind_reverse	1586	1609	.	-	.	gene_id "Dicro61L";
ref_parasitesGeneious	primer_bind_reverse	1709	1728	.	-	.	gene_id "Fas2L";
ref_parasitesGeneious	primer_bind_reverse	1843	1863	.	-	.	gene_id "Fas3L";
ref_parasitesGeneious	primer_bind_reverse	1950	1969	.	-	.	gene_id "Entero2L";
ref_parasitesGeneious	primer_bind_reverse	2051	2072	.	-	.	gene_id "Entero4L";
```

4. Analyses script

S4 File. aMPlexmpileup.sh

#Before lauching the script, the bam files from the Ion Torrent can be renamed with a more convenient name:
ls -l *.{bam,bai} | awk '{print $9}' > oldNames.txt
#Open the oldNames.txt in a text editor, (eventually in Excel) and type in a second column (or separated by a tab) the new names for each file, with a .bam or .bai (as appropriate) extension in the file name.
#Choose the name according to your objective, usually we use the sample name and replicate number.
Create a text file with the two columns: RenameOldNew.txt Be careful to have Linux type Line Feed, in particular, change them if you have been using Excel
Change both BAM and bai file names with the following command
cat RenameOldNew.txt | while read old new; do mv $old $new; done

When copying the script, the fasta and the gff files from this Word document, it is essential to use the Linux way of handling the newline instruction (use either NotePad++ or the dos2unix command)

#Save the script starting from the following line and name it as aMPlexmpileup.sh
! /bin/bash

Script to analyze amplicons from the aMPlex Torrent workflow
It uses as input files the BAM files resulting from the mapping to the reference sequence with the Ion Torrent Mapping Alignment Program (TMAP). It is necessary to use a fasta file with the same name in the first line as that used for mapping.
It requires in the working directory fasta and gff files from the reference sequence, with each amplicon being named using the attribute misc_feature. It also requires the software featureCounts, samtools, bcftools, and VarScan.
A symbolic link to the VarScan.v2.3.7.jar should be in the same folder.
Modify as desired within the script the names of the fasta and gff files (highlighted).
Run the script as follows to keep a log file (here Report_aMPlex_script.txt) with standard error and standard output together: sh aMPlexmpileup.sh >>Report_aMPlex_script.txt 2>&1

Create a symbolic link to the VarScan jar file using the proper address where you stored it (here ~/Software/VarScan.v2.3.7/VarScan.v2.3.7.jar)
ln -s ~/Software/VarScan.v2.3.7/VarScan.v2.3.7.jar ./VarScan.v2.3.7.jar

Convert BAM to SAM
for file in *.bam
do
echo $file
samtools view -h $file > $file".sam"
done

Clean extension tags
for file in *.bam.sam
do
mv "$file" "${file%.bam.sam}.sam"
done

Determine the number of reads per amplicon
featureCounts -a ref_parasites.gff -t misc_feature -R -o CountAmplicons.count *.sam

Determine the number of reads that includes the primers (to assess primer dimers (by subtracting the number of Amplicon reads) and other artefacts)
featureCounts -a ref_parasites.gff -t primer_bind -R -o CountUpperPrimers.count *.sam
featureCounts -a ref_parasites.gff -t primer_bind_reverse -R -o CountLowerPrimers.count *.sam

```
# Identify variants. These lines identify the variants with each sample analyzed in a sequencing run in a different file.
# Mpileup generates a pileup of read bases using the alignments to the reference sequence.
# Varscan detects variation based on the reference sequence and produces a table with the percentage of reads with each SNP.
# To permit species identification from the varscan files, discriminative SNP in the reference sequence should be indicated using the degeneracy IUPAC code (e.g., Y, R, W, ...).

for file in *.bam
do
samtools mpileup -f ref_parasites.fasta $file | java -jar VarScan.v2.3.7.jar mpileup2snp >$file.varscan
done

 # Clean extension tags
for file in *.bam.varscan
do
mv "$file" "${file%.bam.varscan}.varscan"
done

# Produce consensus sequences in the fastq format, assuming that the vcfutils.pl script is located in the following path: /usr/share/samtools/vcfutils.pl (or modify script accordingly)
# Because degeneracy in the reference decreases the accuracy of the consensus sequences obtained, a sequence without degeneracy is provided with a different file name (here ref_parasites2.fasta) but with the same name in the first line of the fasta file.

for file in *.bam
do
#use this command if you have installed version 1.x (at least up to 1.2) of samtools and bcftools
samtools mpileup -uf ref_parasites2.fasta $file | bcftools call -c | vcfutils.pl vcf2fq > $file.fq
#If you have an older version of samtools installed (0.1.19 and earlier), you should use this version instead (uncomment it, and comment the previous line)
#samtools mpileup -uRf ref_parasites2.fasta $file | bcftools view -cg - | /usr/share/samtools/vcfutils.pl vcf2fq > $file.fq
done

# Clean up extension tags
for file in *.bam.fq
do
mv "$file" "${file%.bam.fq}.fq"
done

# Clean up files
mkdir samfile
mkdir countread
mkdir Varscan
mkdir Consensus
mkdir featureCount
mkdir BAMs
mv *.sam samfile
mv *.count countread
mv *.summary countread
mv *.varscan Varscan
mv *.fq Consensus
mv *.featureCounts featureCount
```

```
mv *.bam BAMs
mv *.bai BAMs

# Convert the consensus fastq files in the Consensus folder in fasta

# Recover the sequence from the fastq file (single sequence file)
cd Consensus
for file in *.fq
do
sed -r '/\+/q' $file | sed -r 's/\+//g' | sed -r '/@/d' > $file.seq
done

# Clean extension tags
for file in *.fq.seq
do
mv "$file" "${file%.fq.seq}.seq"
done

# Create a fasta file by using the name of the file as the name of the sequence
for file in *.seq
do
echo \>$file | sed 's/.seq//' >tempfile
cat $file >>tempfile
mv tempfile $file.fa
done

# Clean extension tags
for file in *.seq.fa
do
mv "$file" "${file%.seq.fa}.fa"
done

# Final clean up
rm *.seq
mkdir Fasta
mv *.fa Fasta
```

Genus	species	Diagnostic SNP
Taenia	*solium*	74:A, 80:C, 85:T, 86:G, 207:G, 220:G, 229:T, 236:A, 241:A, 250:G, 259:A
	saginata	74:G, 80:T, 85:A, 86:T, 207:A, 220:A, 229:A, 236:G, 241:G, 250:A, 259:T
	asiatica	74:A, 80:T, 85:A, 86:T, 207:A, 220:A, 229:A, 236:G, 241:G, 250:A, 259:T
Echinococcus	*granulosus*	562:A,C,T
	multilocularis	562:G
Diphyllobothrium	*latum*	671:G, 781:T, 784:T, 787: A, 793: A
	dendriticum	671:A, 781:C, 784:C, 787: G, 793: T
	nihonkaiense	671:A, 781:T, 784:C, 787: A, 793: T
Dicrocoelium	*dendriticum*	1578:G
	chinensis	1578:A
Fasciola	*hepatica*	1696:A, 1697:T, 1815:C
	gigantica	1696:G, 1697:C, 1815:T

S4 Table. Location of the diagnostic SNPs for species discrimination to interpret the varscan data files when using the ref_parasites fasta file for mapping the reads.

S5 File. *Ascaris* and *Trichuris* sequences

>Ascaris_Asc4_Most_Ancient_samples
GCGTATTGGYCCTAATAAGGTTAGTTTTATAGGTTTTTTGCAGGCCATTTTTGATGGTGTTAAACTTTTAAAGAA
GGAGCAGATGACTCCTYTGAATTCTTCGG
>Ascaris_Asc4_Sample_H
GCGTATTGGYCCTAATAAGGTTAGTTTTATAGGTTTTTTGCAGGCTATTTTTGATGGTGTTAAACTTTTGAAGAA
GGAGCAGATGACTCCTYTGAATTCTTCGG
>Ascaris_Asc4_Sample_Na
GCGTATTGGYCCTAATAAGGTTAGTTTTATGGGTTTTTTGCAGGCTATTTTCGATGGTGTTAAACTTTTGAAGAA
GGAGCAGATGACTCCTYTGAATTCTTCGG
>Trichuris_Trich3-4_Major_ancient_European_Sequence
TCATCCAAATGATTGATTATGACCTCGATGTTGAATCATTTGCACACATAGTTTGCAGAAGAATTTTATTCTCGT
CTGTTCGACGATAGACATTACGTGATTTGAGTTTAAACC
>Trichuris_Trich3-4_Minor_ancient_European_Sequence_v1
TCATCCAAATGATTGATTATGACCTCGATGTTGAATCATTTGTACACATAGTTTGCAGAAGAATTTTATTCTCGT
CTGTTCGACGATAGACATTACGTGATTTGAGTTTAAACC
>Trichuris_Trich3-4_Minor_ancient_European_Sequence_v2
TCATCCAAATGATTGATTATGACCTCGATGTTGAATCATTTGTACATATAGTTTGCAGAAGAATTTTATTCTCGT
CTGTTCGACGATAGACATTACGTGATTTGAGTTTAAACC
