# Supplementary material for: A New High-Throughput Approach to Genotype Ancient Human Gastrointestinal Parasites
Source: PLoS One. 2016 Jan 11;11(1):e0146230. doi: 10.1371/journal.pone.0146230 (PMC4709038; doi:10.1371/journal.pone.0146230)
Supplement: S1 Table — (DOCX) [file pone.0146230.s009.docx]

| **Primer name** | ***Organism***  **Gene** | **Size of amplicon (bp)** | **Primer sequences** |
| --- | --- | --- | --- |
| Tae23 | *Taenia*  Cytochrome oxydase 1 (cox1) | 73 | U : GAG GTT TTA GGT TCW TAT GGT T  L : CAT TAT GAG AAG CYA CAG GAC T |
| Tae32 | *Taenia*  Cytochrome b (cytb) | 113 | U : ATA TGG CTC GTG CTT TGT ATT ATT C  L: AGG TAA TAT ATA TCC WGT AAA AGC CTC |
| Echino5 | *Echinococcus*  Phosphoenolpyruvate carboxykinase (pepck, nuclear) | 109 | U : CAC ATG TTG CTG AAG GCG TTA  L : AAT GTA CGG TCG TTC ATG CAG |
| Echino23 | *Echinococcus*  cox1 | 99 | U: TTT TGA TCC GTT AGG TGG T  L: CCA AAT CCA GGC ARA ATC |
| Diphyllo2 | *Diphyllobotrium*  cox1 | 52 | U: GTT GTG TGG GGG CAT CAT A  L:CAG CCG TCT TTA CAT CTA AAC C |
| Diphyllo23 | *Diphyllobotrium*  cox1 | 67 | U: TTT AYG GGT TGT TAT TTG CT  L: ATA TGA TGC CCC CAC ACA |
| Asc2 | *Ascaris*  cytb | 74 | U: GCC AAA GCA CCA TCA TTA GAA TA  L : GGT ATG GTT TTG GGT TTT CAG A |
| Asc4 | *Ascaris*  NADH dehydrogenase 1 (nad1) | 104 | U : GCG TAT TGG YCC TAA TAA GGT TAG T  L :CCG AAG AAT TCA RAG GAG TCA |
| Trich3 | *Trichuris trichiura*  Large ribosomal subunit (LSU) | 74 | U :TCA TCC AAA TGA TTG ATT ATG ACC T  L :CGA AAA TAA AGT TCT TCT GCA AAC TA |
| Trich4 | *Trichuris trichiura*  LSU | 91 | U: TCG ATG TTG AAT CAT TTG TAT ATA TAG T  L:GGT TTA AAC TCA AAT CAC GTA ATG T |
| Dicro22 | *Dicrocoelium*  Internal transcribed spacer 2 (nuclear) | 76 | U : TAC ACA CAC CTA GTT ATC AGA CAG  L: ACA GAC CGC GCA TAA ATA |
| Dicro6.1 | *Dicrocoelium*  nad1 | 77 | U: TAA GTA TAA GTT TST GGT TTC TCA GTT TC  L: AAG CWA CCA AAA TCA TCA AAA ACA |
| Fas2 | *Fasciola*  cox1 | 69 | U : GTT GAT TGG GGG KTT TGG TA  L : CGA GGC AAA TTC AAA TCA GG |
| Fas3 | *Fasciola*  rRNA18s (nuclear) | 85 | U : AAC CTG CGG AAG GAT CAT TA  L: GCA AAT TTT TAT CGC ATG ACA |
| Entero2 | *Enterobius vermicularis*  SL1RNA (nuclear) | 56 | U: TTT ATT TCC AAG CCA CAG ACT CA  L: AAT TTC TCG TTC CGG CTC AG |
| Entero4 | *Enterobius vermicularis*  cox1 | 53 | U: CTG TGC CRA CTG GGG TAA AG  L : TCC CCC TAT CAA AGT CAA CAA C |
