# Supplementary material for: A New High-Throughput Approach to Genotype Ancient Human Gastrointestinal Parasites
Source: PLoS One. 2016 Jan 11;11(1):e0146230. doi: 10.1371/journal.pone.0146230 (PMC4709038; doi:10.1371/journal.pone.0146230)
Supplement: S2 Table — When several related sequences were available, only the positions differing with the reference sequence are indicated, whereas identical positions are represented by dots. The IUPAC nucleotide ambiguity code was used. (DOCX) [file pone.0146230.s010.docx]

| **Genus** | **species** | **PCR product 1** | **PCR product 2** |
| --- | --- | --- | --- |
| *Taenia* | *solium* | **Tae23** **CA**T**C**TAG**C**TGTT**TG**GT**GA**ATTTTTTTATG T............................ .................G........... | **Tae32** AAGT**T**A**T**A**G**AAAGAAGGGTGT**G**TGAAATGT**T**GG**G**TTT**A**TTTT**A**TATTTATT**GG**TTATGGG**A**G ....................................................C......... .................................A..................G......... |
|  | *saginata* | .G.....T....AT..RG........... .G.A...T....AT...G........... | ........A............A........A......G....G........A........T. |
|  | *asiatica* | .......T....AT...G........... | ........A............A........A......G....G........A........T. |
| *Echino-coccus* | *granulosus* | **Echino5** AAGGCATCCTTGGCCAATGGATCTCGCCAAAAGATCTTGACGAATCCCTTAGGGATCGCTTCCCTGG | **Echino23** GGTGATCCTATTTT**G**TTTCAACATATGTT**T**TGGTTTTTTGG**C**CATCC**T**GAGGTTTATGTG**T**T ..............A................................H.............. ..............A..............Y...........T.....T.............. ..............A.............................................C. |
|  | *multilo-cularis* | **...................................................................** | ............Y.A..........................T.....G...........T.. ..............R..........................T.....G...........G.. |
| *Diphyllo-bothrium* | *latum* | **Diphyllo2** TGTTTAC**G**GT**G** | **Diphyllo23** ATGTT**T**TCTATAGT**T**TG**T**TT**A**GGTAG**A**GT |
|  | *dendri-ticum* | .......**A**..**A** | .....Y........C..C..G.....T.. |
|  | *nihon-kaiense* | **.......A...** | .................C........T.. |
| *Ascaris* | *lumbri-coides and suum* | **Asc2** TTTTGACTGGTA**CT**TTTTTG**G**CTTTTTAT ............**GT**......**R**........ ............**AC**..... **G**........ | **Asc4** TTTATAGGTTT**T**TTGCAGGC**C**ATTTTTGATGGTGTTAAACTTTTAAA**G**AA**R**GAGCAGA ...........**Y**........**T**..........................**A**..**G.......** |
| *Trichuris* | *trichiura* | **Trich3** CGATGTTGAATCATTTGTATATA | **Trich4** TTGCAGAAGAACTTTATTTTCGTCTGTTCGACGATGGA |
| *Dicro-coelium* | *dendri-ticum* | **Dicro22** GTGGAGATGTGTCTACGGAGTCGTGGCT | **Dicro6**.1 GGGATTGGTTGTCTTG**G**GTTGGTT |
|  | *chinensis* | ............................ | ................**A**....... |
| *Fasciola* | *hepatica* | **Fas2** ATTATTTGTTGCC**YT**TATTGTTAGG**T**ATT .............CY..........G... | **Fas3** CCTGAAAATCTACTCT**C**ACACAAGCGATACACGTGTGACCGTCA |
|  | *gigantica* | .Y.....A.....T..GC.T..G...... .............T..GC.T..G...... .......A..A..T..GC.T......... | ................**T**........................... |
| *Entero-bius* | *vermicu-laris* | **Entero2** CTGATGTTCATGT | **Entero4** GTTTTTAGTTG |
