# Supplementary material for: A New High-Throughput Approach to Genotype Ancient Human Gastrointestinal Parasites
Source: PLoS One. 2016 Jan 11;11(1):e0146230. doi: 10.1371/journal.pone.0146230 (PMC4709038; doi:10.1371/journal.pone.0146230)
Supplement: S3 Table — Optimal MgCl2, and primer concentrations used for each individual multiplex PCR are shown. (DOCX) [file pone.0146230.s011.docx]

| **Multiplex 1**  4 mM MgCl_2_ | | **Multiplex 2**  5 mM MgCl_2_ | | **Multiplex 3**  4mM MgCl_2_ | | **Multiplex 4**  5 mM MgCl_2_ | |
| --- | --- | --- | --- | --- | --- | --- | --- |
| primers | [µM] | primers | [µM] | primers | [µM] | primers | [µM] |
| Tae32 | 0.15 | Tae23 | 0.2 | Trich3 | 0.25 | Dicro6.1 | 0.2 |
| Echino5 | 0.08 | Trich4 | 0.08 | Entero4 | 0.08 | Asc4 | 0.2 |
| Diphyllo2 | 0.08 | Dicro22 | 0.08 |  | | Fas3 | 0.08 |
|  | | Entero2 | 0.08 |  |  | Fas2 | 0.08 |
|  |  | Diphyllo23 | 0.08 |  |  | Echino23 | 0.08 |
|  |  |  | |  |  | Asc2 | 0.08 |
