# Supplementary material for: A New High-Throughput Approach to Genotype Ancient Human Gastrointestinal Parasites
Source: PLoS One. 2016 Jan 11;11(1):e0146230. doi: 10.1371/journal.pone.0146230 (PMC4709038; doi:10.1371/journal.pone.0146230)
Supplement: S4 Table — (DOCX) [file pone.0146230.s012.docx]

| **Genus** | **species** | **Diagnostic SNP** |
| --- | --- | --- |
| *Taenia* | *solium* | 74:A, 80:C, 85:T, 86:G, 207:G, 220:G, 229:T, 236:A, 241:A, 250:G, 259:A |
|  | *saginata* | 74:G, 80:T, 85:A, 86:T, 207:A, 220:A, 229:A, 236:G, 241:G, 250:A, 259:T |
|  | *asiatica* | 74:A, 80:T, 85:A, 86:T, 207:A, 220:A, 229:A, 236:G, 241:G, 250:A, 259:T |
| *Echinococcus* | *granulosus* | 562:A,C,T |
|  | *multilocularis* | 562:G |
| *Diphyllobothrium* | *latum* | 671:G, 781:T, 784:T, 787: A, 793: A |
|  | *dendriticum* | 671:A, 781:C, 784:C, 787: G, 793: T |
|  | *nihonkaiense* | 671:A, 781:T, 784:C, 787: A, 793: T |
| *Dicrocoelium* | *dendriticum* | 1578:G |
|  | *chinensis* | 1578:A |
| *Fasciola* | *hepatica* | 1696:A, 1697:T, 1815:C |
|  | *gigantica* | 1696:G, 1697:C, 1815:T |
